# Expeditious synthesis and preliminary antimicrobial activity of deflazacort and its precursors†

**DOI:** 10.1039/c9ra03673c

**Published:** 2019-07-10

**Authors:** Anna Esposito, Eliana De Gregorio, Maria De Fenza, Daniele D'Alonzo, Anil Satawani, Annalisa Guaragna

**Affiliations:** Department of Chemical Sciences, University of Napoli Federico II Via Cintia 80126 Napoli Italy annalisa.guaragna@unina.it; Department of Molecular Medicine and Medical Biotechnologies Via S. Pansini, 5 80131 Napoli Italy; Symbiotec Pharmalab Pvt Ltd Pithampur Indore India

## Abstract

The synthesis of deflazacort (DFZ) and a preliminary evaluation of its microbial activity against the human pathogens *Acinetobacter baumannii* and *Staphylococcus aureus* is herein reported. While DFZ is inactive, one of its synthetic precursors showed a strong antibacterial activity against both Gram-negative and -positive bacteria.

## Abstract 2

Synthesis of deflazacort: unexpected antibacterial activity of its epoxide synthetic precursor.

## Graphical abstract

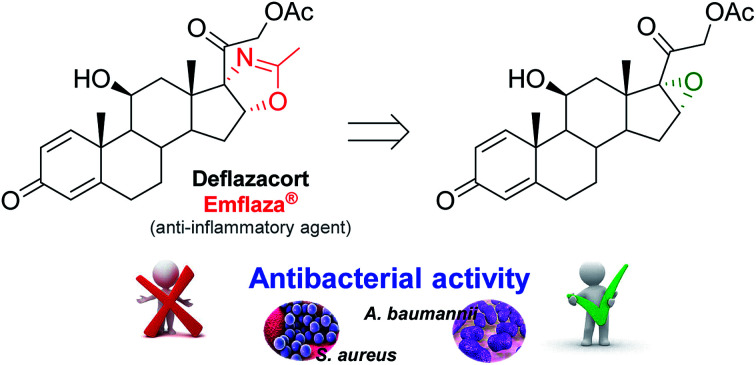

## Introduction

Mineralocorticoids (MCs) and glucocorticoids (GCs) belong to the group of corticosteroids (hormones with 21 carbon atoms) and are basically classified based on their preferential biological activity, depending on their involvement in the balancing of fluids and electrolytes or in the regulation of carbohydrate metabolism, respectively.^1^

**Fig. 1:**
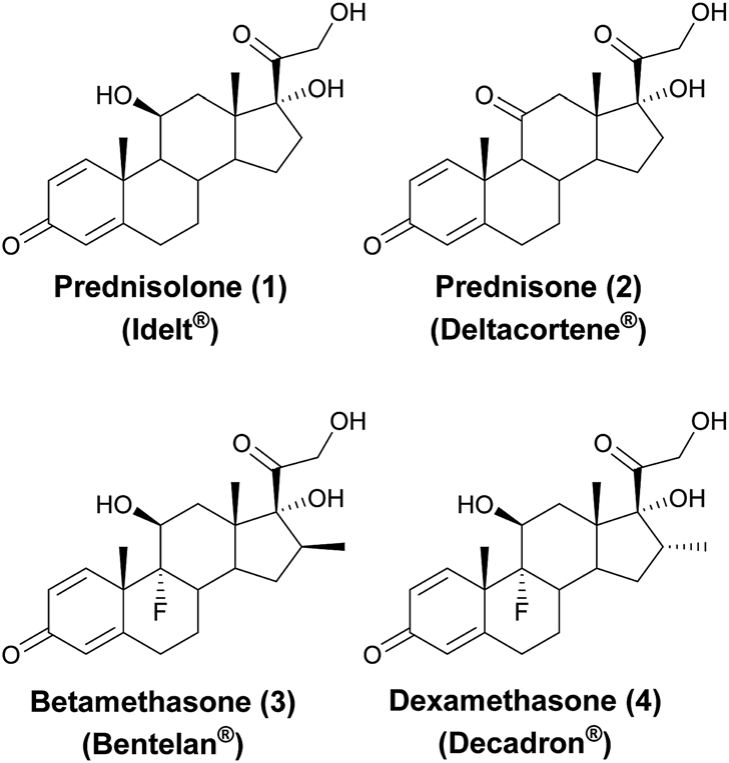
Anti-inflammatory man-made glucocorticoids.

The immunosuppressive and anti-inflammatory properties of GCs, mainly due to inhibition of specific leucocyte functions, are well-established.^2^ The first efforts in this field led to the synthesis of novel corticosteroids such as prednisolone (1, Fig. 1) and prednisone (2, Fig. 1), which were endowed with a higher anti-inflammatory potency than natural cortisone, along with lower undesired effects. Inspired by these results, a variety of other corticosteroids with different therapeutic applications were synthetized, including fluorinated GCs betamethasone (3) and dexamethasone (4).

**Fig. 2:**
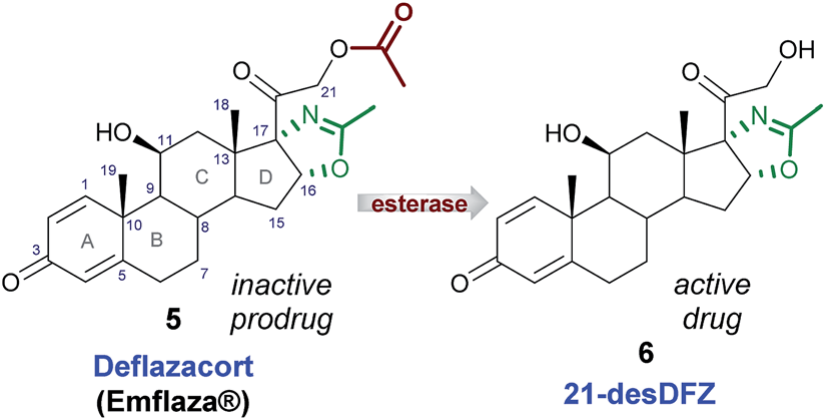
Deflazacort (5) and its active metabolite 21-desDFZ (6).

The search of novel anti-inflammatory drugs characterized by high efficacy and good tolerability in each age group, led to identification of deflazacort (5), a heterocyclic corticosteroid, acting as a new anti-inflammatory/immunosuppressive agent. Indeed, 5 is endowed with a lower interference with carbohydrate and phosphocalcium metabolism compared with previous-generation corticosteroids and it is therefore able to produce less serious metabolic events.^3,4^ DFZ, the oxazoline-derivative prodrug of prednisolone,^5^ after oral administration is rapidly converted into the active metabolite, 21-desacetyldeflazacort (6, Fig. 2), by cellular esterases.^6^

To exert its pharmacological effect, DFZ acts as corticosteroid hormone receptor agonist, exploiting the high binding affinity for tissue glucocorticoid receptors.^7^ Very recently, DFZ was approved^8^ by the FDA (trade name: Emflaza) for its use in treatment of Duchenne Muscular Dystrophy (DMD), a genetic disorder that affects 1/3600 infants worldwide. However, the precise mechanism by which DFZ exerts its therapeutic effect in patients with DMD is still unknown.

Even though the anti-inflammatory and immunosuppressive activity^4^ of DFZ is well-known, there are no reports to the best of our knowledge about its direct action on bacterial infections. In this context, our ongoing interest into the synthesis of bioactive compounds^9^ prompted us to develop a new synthetic protocol for the preparation of DFZ. In addition, the evaluation of the antimicrobial potential of DFZ as well as of its steroidal synthetic precursors against *Acinetobacter baumannii* and *Staphylococcus aureus* was assessed by *in vitro* assays.

## Results and discussion

**Scheme 1:**
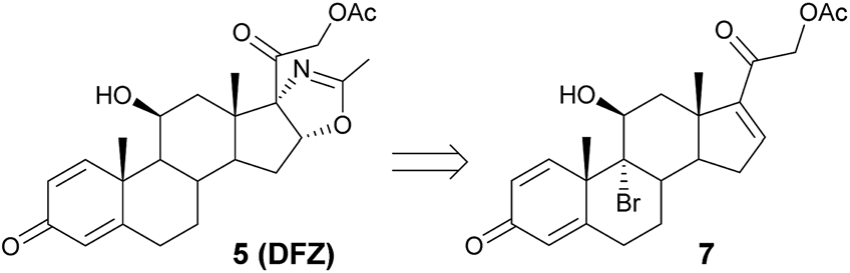
Retrosynthetic pathway to DFZ synthesis.

Our synthesis started from 9-bromotriene acetate 7 (Scheme 1), readily obtained from the commercially available tetraene acetate^10^ (3TR), an industrial key intermediate already used for the synthesis of currently marketed bioactive glucocorticoid drugs.^11^

The use of 7 allowed us to have the proper functionalization at C21 position, as required by the DFZ structure, avoiding the installation of the acetylated 21-hydroxyl function in a late stage of the synthesis, as commonly reported for oxazoline-containing steroids.^12^

At first, double bond epoxidation at C16–C17 positions of the starting material was studied, to be then further elaborated for conversion into the oxazoline moiety of 5. The epoxidation reaction was attempted on both 7 and its debrominated derivative 8, in turn obtained by treatment of 7 with Bu_3_SnH and AIBN in refluxing THF (93–98% yield) (Table 1).

**Table 1:** Epoxidation of derivatives 7 and 8^a^

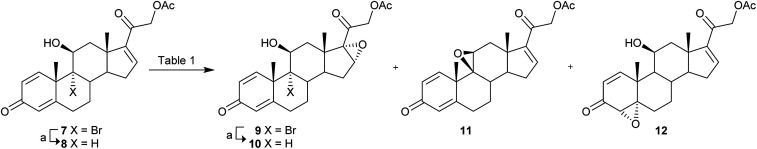						
Entry	Compd	Reacting system	Solvent	Temp [°C]	*t* [h]	Yield [%]
1	7	MCPBA	DCM	Δ	16	9: 47
2	7	Oxone/NaHCO_3_/aq. Na_2_EDTA	CF_3_COCH_3_/ACN	rt	18	11: 51
3	7	H_2_O_2_/NH_4_HCO_3_	ACN/H_2_O	rt	16	11: 59
4	8	MCPBA	DCM	Δ	16	10: 40
5	8	Oxone/NaHCO_3_/aq. Na_2_EDTA	CF_3_COCH_3_/ACN	rt	18	12: 55
6	8	H_2_O_2_/NH_4_HCO_3_	ACN/H_2_O	rt	18	—

a Bu_3_SnH, AIBN, THF, reflux, 30′, 93–98%.

Nearly neutral or acidic conditions were tested, since alkaline double bond epoxidation was hampered by the presence of the acetyl group at C21 position. The best results were obtained by treatment of 7 or 8 with *m*CPBA in refluxing DCM (entries 1 and 4, Table 1), obtaining in both cases the desired α-epoxides 9 and 10 in 47% and 40% yield. On the contrary, the use of *in situ* generated TFDO (Oxone/NaHCO_3_/aq. Na_2_EDTA) (entries 2 and 5, Table 1) demonstrated to be unsuited, as it led to the formation of undesired epoxides 11 and 12 (from 7 and 8, respectively). The use of bicarbonate-catalyzed epoxidation with aqueous hydrogen peroxide,^13^ at near neutral pH, afforded only a minor amount of 9 (13%, from 7), while epoxide 11 was isolated as the main product (59%) (entry 3, Table 1). Under the same conditions, the reaction from olefin 8 led to the recovery of totally unreacted starting material (entry 6, Table 1).

**Scheme 2:**
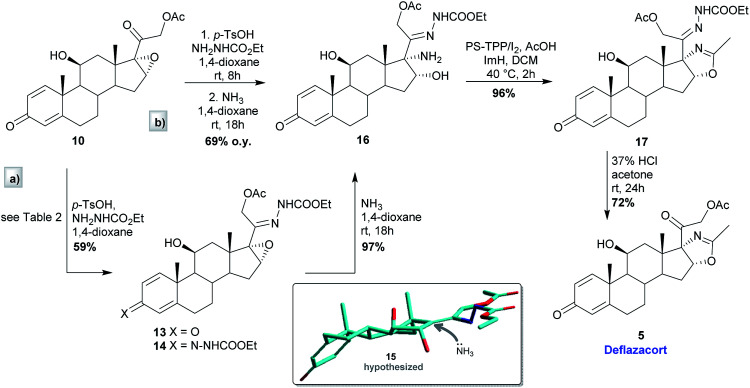
Synthetic steps to deflazacort from epoxide 10.

With epoxide 10 in hand, conversion of the latter into *cis*-17-amino-16-hydroxy derivative 16 was accomplished either by a stepwise route (path a, Scheme 2) or by a one-pot procedure (path b, Scheme 2), exploiting the formation of the corresponding carboethoxy hydrazone 13. Indeed, hydrazone groups-containing oxidosteroids are reported to be able to efficiently drive the regio- and stereoselective ring opening of neighbouring three-membered heterocycles.^14^ Thus, hydrazone 13 could represent a valuable intermediate to selectively introduce a vicinal amino-alcohol function in C16 and C17 positions with the desired *cis*-configuration.

As shown in Table 2, different reaction conditions were explored, treating 10 with ethyl carbazate, varying both the reaction solvent and the activating agent. In almost all cases, unsatisfactory results were obtained (entries 1–3), since only limited conversions were achieved, while the recovery of unreacted staring material was mainly found. On the other hand, when the reaction was carried out with Py·HCl in pyridine (entry 4), the protection of both C20 and C3 carbonyl functions occurred, affording hydrazone 14 in 30% yield. The desired C20 monoprotected derivative 13 was instead easily obtained in good yield (59%), using ethyl carbazate and *p*-toluenesulfonic acid in anhydrous 1,4 dioxane (entry 5).

**Table 2:** Experimental conditions for the conversion of ketone 10 into carboethoxy hydrazone 13

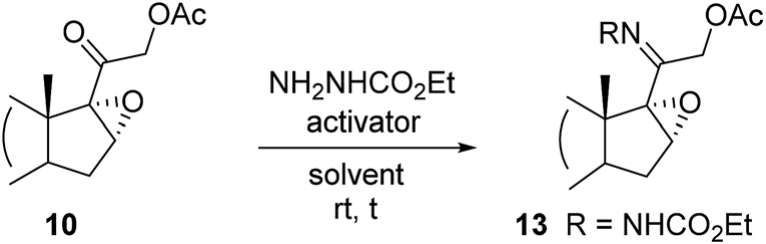				
Entry	Activating agent	Solvent	*t* (h)	Yield (%)
1	AcOH	1,4-Dioxane	18	12
2	H_2_SO_4_	DMF	16	20
3	CSA	Toluene	16	16
4	Py-HCl	Pyridine	24	30
5	*p*-TsOH	1,4-Dioxane	8 h	59

The epoxide ring opening from hydrazone 13 was then considered. Treatment of 13 with NH_3_ in dioxane afforded the desired *cis*-17-amino-16-hydroxy derivative 16 in 97% yield (Scheme 2, path a). Differently from the well-known acid-catalysed epoxide ring opening carried out on similar steroid derivatives, the stereochemical outcome of the reaction under basic conditions can be rationalized assuming that NH_3_ deprotonates hydrazone 13, enabling epoxide ring opening to give the intermediate 15 (PM3; HyperChem™, rel 8.0.3). The latter then undergoes nucleophilic attack by ammonia from the less hindered side of ring D, leading to amino-alcohol 16. As previously mentioned, C20 protection and epoxide ring opening steps were also performed in a one-pot procedure (Scheme 2, path b) leading to the same results in terms of reaction times and yields.

Oxazoline ring formation from 16 was eventually studied, using the well-known polymer supported triphenylphosphine/iodine/imidazole^15^ (PS-DPP/I_2_/ImH) system as the activating agent.^16^ Treatment of AcOH with 2.0 equivalents of the previously mixed PS-DPP/I_2_ followed by the addition of 16 and ImH to the reaction mixture provided, already after 2 h in refluxing DCM, the desired 17 in high yield (96%) and purity. The first equivalent of PS-TPP/I_2_ is needed to activate acetic acid, whereas a second equivalent of the complex promotes the hydroxyl group activation. The addition of ImH to the reaction medium ensures neutral/mild alkaline environment by trapping protons released during the course of the reaction and the amide proton abstraction, thus promoting oxazoline ring closure. It's worth noting that the only reaction by-product, *i.e.* the resin-bound phosphine oxide, can be easily filtered off and eventually recycled, providing the original phosphine by reduction with trichlorosilane.^17^

Lastly, removal of C20 hydrazone function (HCl/acetone) on 17 smoothly provided the pure Deflazacort (5) in 72% yield.

DFZ and its synthetic precursors were evaluated in experiments aimed to determine the minimum inhibitory concentration (MIC) against *Staphylococcus aureus* and *Acinetobacter baumannii*,^18^ two bacterial pathogens that have become one of major causes of life-threatening infections. *A. baumannii* and *S. aureus* were classified as critical and high priority pathogens, respectively, as they rapidly developed multi-resistance to antibiotics.^19^ Compounds 5 and 16 were inactive as growth inhibitors even at the high experimental concentrations (1 mg mL^−1^), whereas compounds 13 and 17 showed only weak antimicrobial activity (Table 3). On the contrary, epoxide 10 showed inhibition at concentrations as low as 16 μg mL^−1^ for both *S. aureus* and *A. baumannii* strains. Interestingly, compounds 10 and 13 also work as bactericidal agents, having minimum bactericidal concentration (MBC) values generally equal or three-fold higher than their MIC values (Table 3).

**Table 3:** MIC (mg mL^−1^) and MBC (mg mL^−1^) values of DFZ and its synthetic precursors on one reference strain of *S. aureus* and *A. baumannii* compared with MIC of some known antimicrobial agents^a^

Compound	Bacteria			
	*S. aureus* ATCC 29213		*A. baumannii* ATCC 17978	
	MIC	MBC	MIC	MBC
10	0.016	0.016	0.016	0.016
13	0.375	1	>1	>1
16	>1	>1	>1	>1
17	0.75	>1	>1	>1
DFZ	>1	>1	>1	>1
Vancomycin	0.001	ND	ND	ND
Methicillin	0.0025	ND	ND	ND
Gentamicin	0.0005	ND	0.0015	ND
Ampicillin	ND	ND	>0.032	ND
Amikacin	ND	ND	0.004	ND

a MIC experiments were carried out in triplicates and was determined by broth microdilution assay as previously describes;^20^ ND: not determined.

Cytotoxicity of 10 was assessed *in vitro* using hemolytic assay measuring its hemolytic activity against horse RBCs at final concentrations ranging from 1 to 512 μg mL^−1^. The glucocorticoid epoxide 10 showed no hemolytic activity at concentrations of up to 128 μg mL^−1^, whereas was 62.8% and 78.6% at 256 μg mL^−1^ and 512 μg mL^−1^, respectively.^21^

## Conclusions

In summary, we tuned up a novel and efficient synthesis of deflazacort in few steps from 9-bromotriene acetate 7 by regio- and stereoselective functionalization of C16 and C17 positions. In search for antibacterial activity of DFZ and its synthetic precursors, we found that epoxide 10 displayed interesting biological properties. Further studies will be aimed to an in-depth evaluation of the antimicrobial properties of 10, as well as the determination of the mechanism of action.

## Experimental

### Chemical synthesis

#### General methods and materials

All chemicals and solvents were purchased with the highest degree of purity (Sigma-Aldrich, Alfa Aesar, VWR) and used without further purification. 9-Bromotriene acetate 7 was provided by Symbiotec Pharmalab PVT. All moisture-sensitive reactions were performed under nitrogen atmosphere using oven-dried glassware. The reactions were monitored by TLC (precoated silica gel plate F254, Merck) and the products were detected by exposure to ultraviolet radiation, iodine vapor, and chromic mixture. Column chromatography: Merck Kieselgel 60 (70–230 mesh); flash chromatography: Merck Kieselgel 60 (230–400 mesh). The purity of the synthetic intermediates and the final compound was determined by CHNS analysis and was ≥95% in all cases. NMR spectra were recorded on NMR spectrometers operating at 400 MHz (Bruker DRX, Bruker AVANCE) or 500 MHz (Varian Inova), using CDCl_3_ solutions unless otherwise specified. Coupling constant values (*J*) were reported in Hz.

#### Synthetic procedures

##### 1,4-Pregnadiene-9-bromo-11-hydroxy-16α,17α-epoxy-3,20-dione (9)

To a stirred solution of 9-bromotriene acetate 7 (1.0 g, 2.16 mmol) in anhydrous CH_2_Cl_2_ (30 mL), *m*-CPBA (0.75 g, 4.32 mmol) was added at room temperature. The mixture was warmed to reflux and stirred for 16 h. Then, aq. NaHCO_3_ was added and the mixture extracted with CH_2_Cl_2_; the organic layer was dried (Na_2_SO_4_) and the solvent evaporated under reduced pressure. The solid residue was recrystallized from AcOEt to give the final compound 9 (0.49 g, 47% yield) as a white solid. ^1^H NMR (400 MHz): *δ* 1.41 (s, 3H), 1.48–1.55 (m, 1H), 1.70 (s, 3H), 1.71–1.82 (m, 2H), 1.94–2.05 (m, 3H), 2.15 (s, 3H), 2.16–2.25 (m, 1H), 2.36–2.49 (m, 2H), 2.55–2.68 (m, 1H), 3.85 (s, 1H), 4.59 (d, *J* = 13.4, 1H), 4.67 (d, *J* = 13.4, 1H), 4.76 (bs, 1H), 6.07 (bs, 1H), 6.32 (d, *J* = 10.1, 1H), 7.21 (d, *J* = 10.1, 1H). ^13^C NMR (100 MHz): 18.3, 20.4, 24.9, 27.0, 28.3, 30.4, 33.5, 37.3, 39.3, 42.1, 50.2, 61.1, 65.7, 70.5, 75.9, 85.3, 125.1, 129.3, 152.3, 165.5, 170.4, 186.2, 198.9. Anal. calcd for C_23_H_27_BrO_6_: C, 57.63; H, 5.68; Br, 16.67. Found: C, 57.75; H, 5.66; Br, 16.62.

##### 1,4-Pregnadiene-11-hydroxy-16α,17α-epoxy-3,20-dione (10)

To a boiling and stirring suspension of epoxide 9 (0.50 g, 1.04 mmol) in anhydrous THF (13.0 mL), a solution of Bu_3_SnH (0.36 mL, 1.25 mmol) and AIBN (catalytic amount, 33.3 mg, 0.21 mmol) in THF (10 mL) was added dropwise under nitrogen atmosphere. The reaction mixture was stirred for 30 min at reflux temperature. The crude residue was then concentrated under reduced pressure and recrystallized from AcOEt to give 10 (0.41 g, 98% yield). Epoxide 10 (40% yield) was also obtained from compound 8 by epoxidation reaction using the same procedure reported for 9. Data for 10: white solid, ^1^H NMR (400 MHz): *δ* 1.02–1.14 (m, 2H), 1.39 (s, 3H), 1.46 (s, 3H), 1.46–1.51 (m, 2H), 1.62–1.70 (m, 1H), 1.97–2.02 (m, 3H), 2.15 (s, 3H), 2.15–2.19 (m, 1H), 2.29–2.38 (m, 1H), 2.51–2.62 (m, 1H), 3.82 (s, 1H), 4.42 (bs, 1H), 4.57 (d, *J* = 13.4, 1H), 4.66 (d, *J* = 13.4, 1H), 6.00 (bs, 1H), 6.26 (dd, *J* = 1.8, 10.1, 1H), 7.21 (d, *J* = 10.1, 1H). ^13^C NMR (100 MHz): 17.4, 20.5, 21.3, 27.8, 28.3, 29.5, 31.9, 33.4, 41.0, 42.0, 44.2, 45.1, 56.2, 61.4, 65.8, 69.9, 122.6, 128.0, 156.1, 169.6, 170.4, 186.6, 190.0. Anal. calcd for C_23_H_28_O_6_: C, 68.98; H, 7.05. Found: C, 69.05; H, 7.03.

##### 1,4,17-Pregnatriene-11-hydroxy-3,20-dione (8)

Compound 8 was obtained in 93% yield from 7 by using the same conditions reported above for preparation of 10. Data for 8: white solid, ^1^H NMR (500 MHz): *δ* 1.06–1.21 (m, 1H), 1.26 (s, 3H), 1.32–1.38 (m, 1H), 1.49 (s, 3H), 1.59–1.63 (m, 2H), 2.06–2.12 (m, 1H), 2.17 (s, 3H), 2.18–2.30 (m, 2H), 2.33–2.43 (m, 2H), 2.45–2.51 (m, 1H), 2.55–2.67 (m, 1H), 4.40 (bs, 1H), 4.85 (d, *J* = 16.1, 1H), 5.01 (d, *J* = 16.1, 1H), 6.01 (bs, 1H), 6.27 (d, *J* = 10.1, 1H), 6.73 (bs, 1H), 7.31 (d, *J* = 10.1, 1H). ^13^C NMR (100 MHz): 18.4, 20.5, 21.2, 30.1, 31.8, 32.8, 33.6, 44.2, 44.6, 46.1, 56.1, 56.4, 65.5, 70.2, 122.5, 128.0, 143.5, 152.2, 156.1, 169.4, 170.2, 186.6, 190.5. Anal. calcd for C_23_H_28_O_5_: C, 71.85; H, 7.34. Found: C, 71.80; H, 7.35.

##### 20-Carboethoxyhydrazone of 1,4-pregnadiene-11-hydroxy-16α,17α-epoxy-3-one (13)

To a stirring suspension of 10 (0.50 g, 1.25 mmol) in anhydrous 1,4-dioxane (24 mL), ethyl carbazate (0.26 g, 2.5 mmol) and *p*-toluenesulfonic acid (0.24 g, 1.25 mmol) were sequentially added at room temperature under argon atmosphere. The resulting mixture was stirred at the same temperature for 8 h. Then aq. NaHCO_3_ was added and the mixture extracted with EtOAc; the organic layer was dried (Na_2_SO_4_) and the solvent evaporated under reduced pressure. The solid residue was recrystallized from Et_2_O to give the final compound 13 (0.36 g, 59% yield) as a white solid. ^1^H NMR (500 MHz): *δ* 1.01–1.12 (m, 2H), 1.15–1.27 (m, 2H), 1.31 (s, 3H), 1.33 (t, *J* = 7.1, 3H), 1.40–1.44 (m, 1H), 1.47 (s, 3H), 1.68 (dd, *J* = 3.1, 14.2, 1H), 1.96–2.04 (m, 2H), 2.13 (s, 3H), 2.15–2.18 (m, 1H), 2.32 (dd, *J* = 3.0, 12.7, 1H), 2.50–2.61 (m, 2H), 3.67 (s, 1H), 4.22–4.29 (m, 2H), 4.31 (d, *J* = 13.1, 1H), 4.40 (bs, 1H), 4.56 (d, *J* = 13.1, 1H), 6.00 (bs, 1H), 6.25 (d, *J* = 10.1, 1H), 7.29 (d, *J* = 10.1, 1H). ^13^C NMR (125 MHz): 14.6, 18.2, 20.6, 21.2, 27.2, 29.9, 31.9, 33.5, 41.4, 41.7, 44.2, 45.9, 55.0, 56.2, 59.6, 62.1, 69.8, 70.2, 122.5, 127.8, 142.5, 153.6, 156.2, 169.7, 171.2, 186.6. Anal. calcd for C_26_H_34_N_2_O_7_: C, 64.18; H, 7.04; N 5.76. Found: C, 64.29; H, 7.02; N 5.75.

##### 20-Carboethoxyhydrazone of 1,4-pregnadiene-16α-amino-11,17α-diol-3-one (16)

Method A. Epoxide 13 (0.30 g, 0.62 mmol) was dissolved in anhydrous 1,4-dioxane (15 mL) under nitrogen atmosphere at rt. Anhydrous ammonia was then gently bubbled into the solution for 3 minutes and the reaction was stirred at the same temperature for 18 h. Nitrogen was bubbled in the reaction mixture until the ammonia was eliminated from the solution (pH = 7). The mixture was diluted with CH_2_Cl_2_ and the organic layers were washed with brine, dried (Na_2_SO_4_) and concentrated under reduced pressure. The solid residue was recrystallized from Et_2_O to give the final compound 16 (0.30 g, 97% yield) as a white solid. Method B (one-pot procedure). To a stirred suspension of 10 (0.50 g, 1.25 mmol) in anhydrous 1,4-dioxane (24 mL), ethyl carbazate (0.26 g, 2.5 mmol) and *p*-toluenesulfonic acid (0.24 g, 1.25 mmol) were sequentially added at room temperature under argon atmosphere. The resulting mixture was stirred at the same temperature for 8 h and then anhydrous ammonia was gently bubbled into the solution for 3 minutes. The reaction mixture was stirred at room temperature for 18 h. The mixture was diluted with CH_2_Cl_2_ and the organic layers washed with brine, dried (Na_2_SO_4_) and concentrated under reduced pressure. The solid residue was recrystallized from Et_2_O to give the final compound 16 (0.43 g, 69% overall yield). ^1^H NMR (400 MHz, acetone-*d*_6_): *δ* 1.04–1.16 (m, 5H), 1.21–1.41 (m, 4H), 1.51 (s, 3H), 1.52–1.57 (m, 1H), 1.78–1.99 (m, 4H), 2.03–2.17 (m, 7H), 2.35 (dd, *J* = 3.4, 13.4, 1H), 2.62 (td, *J* = 4.9, 13.4, 1H), 3.81 (bs, 1H), 4.17 (q, *J* = 7.1, 2H), 4.47 (bs, 1H), 4.76 (d, *J* = 13.0, 1H), 4.90 (d, *J* = 13.0, 1H), 5.16 (dd, *J* = 3.0, 9.2, 1H), 5.92 (bs, 1H), 6.14 (dd, *J* = 1.6, 10.1, 1H), 7.33 (d, *J* = 10.1, 1H). ^13^C NMR (100 MHz, acetone-*d*_6_): 14.0, 18.2, 19.9, 20.8, 29.7, 30.9, 31.6, 33.9, 40.9, 44.0, 46.4, 48.9, 55.6, 58.2, 60.8, 69.4, 81.2, 83.7, 121.8, 127.3, 149.4, 153.6, 155.9, 170.1, 170.3, 185.1. Anal. calcd for C_26_H_37_N_3_O_7_: C, 62.01; H, 7.41; N 8.34. Found: C, 62.08; H, 7.38; N 8.36.

##### 20-Carboethoxyhydrazone of 1,4-pregnadiene-11-hydroxy- 16α,17α-oxazole-3-one (17)

I_2_ (0.30 g, 1.19 mmol) was added to a stirred solution of polymer supported triphenylphosphine (PS-TPP; 100–200 mesh, extent of labeling: ∼3 mmol g^−1^ triphenylphosphine loading) (0.40 g, 1.19 mmol) in anhydrous DCM (20 mL) at rt. Then glacial acetic acid (35 μL, 0.59 mmol) was added and the solution was stirred at room temperature for 20′. Afterwards, 16 (0.30 g, 0.59 mmol) and imidazole (0.16 g, 2.38 mmol) were sequentially added and the solution was warmed to 40 °C and stirred for 2 h. The mixture was then filtered and the solvent removed under reduced pressure. The solid residue was recrystallized from Et_2_O to give the final compound 17 (0.29 g, 96% yield) as a white solid. ^1^H NMR (500 MHz, acetone-*d*_6_): δ 1.04 (dd, *J* = 3.7, 11.4, 1H), 1.07 (s, 1H), 1.09–1.18 (m, 1H), 1.27 (t, *J* = 7.1, 1H), 1.29–1.36 (m, 3H), 1.51 (s, 3H), 1.74 (dd, *J* = 5.7, 13.6, 1H), 1.81 (dd, *J* = 7.6, 13.6, 1H), 1.85–1.90 (m, 4H), 2.12 (s, 3H), 2.18–2.25 (m, 1H), 2.35 (dd, *J* = 3.4, 13.5, 1H), 2.65 (td, *J* = 5.7, 13.5, 1H), 3.83 (bs, 1H), 4.17 (q, *J* = 7.1, 2H), 4.47 (bs, 1H), 4.73 (d, *J* = 13.0, 1H), 4.92 (d, *J* = 13.0, 1H), 5.66 (d, *J* = 5.7, 1H), 5.92 (bs, 1H), 6.14 (dd, *J* = 1.8, 10.1, 1H), 7.31 (d, *J* = 10.1, 1H), 9.37 (bs, 1H). ^13^C NMR (125 MHz, acetone-*d*_6_): 13.2, 14.0, 19.1, 19.9, 20.8, 29.7, 30.6, 31.5, 33.9, 41.7, 43.9, 47.0, 50.4, 55.5, 57.5, 60.9, 69.1, 83.7, 91.6, 121.9, 127.4, 145.9, 153.4, 155.7, 165.1, 169.5, 170.2, 185.0. Anal. calcd for C_28_H_37_N_3_O_7_: C, 63.74; H, 7.07; N 7.96. Found: C, 63.81; H, 7.02; N 7.99.

##### Deflazacort (5)

To a stirred solution of 17 (0.30 g, 0.57 mmol) in acetone (8 mL), HCl 37% solution (0.09 mL, 1.14 mmol) was added and stirring at room temperature for 24 h. Then aq. NaHCO_3_ was added and the mixture extracted with CH_2_Cl_2_; the combined organic layers were washed with brine, dried (Na_2_SO_4_) and the solvent evaporated under reduced pressure. Chromatography of the crude residue over silica gel (hexane : acetone = 7 : 3) gave the pure DFZ (5) (0.18 g, 72% yield) as a white solid. ^1^H NMR (400 MHz, CD_3_OD): *δ* 1.01 (dd, *J* = 3.6, 11.3, 1H), 1.02 (s, 3H), 1.06–1.25 (m, 2H), 1.49 (s, 3H), 1.75 (dd, *J* = 5.9, 13.8, 1H), 1.79–1.85 (m, 1H), 1.89 (dd, *J* = 3.9, 14.2, 1H), 1.97 (s, 3H), 2.01 (dd, *J* = 2.7, 13.8, 1H), 2.06–2.12 (m, 1H), 2.13 (s, 3H), 2.20 (dd, *J* = 4.1, 11.6, 1H), 2.37 (ddd, *J* = 1.8, 4.6, 13.4, 1H), 2.65 (td, *J* = 5.7, 13.4, 1H), 4.41 (dd, *J* = 3.2, 6.3, 1H), 4.93 (s, 2H), 5.30 (d, *J* = 5.5, 1H), 6.00 (bs, 1H), 6.25 (dd, *J* = 1.9, 10.1, 1H), 7.45 (d, *J* = 10.1, 1H). ^13^C NMR (125 MHz, CD_3_OD): 12.5, 17.2, 18.9, 20.1, 30.4, 31.6, 33.9, 34.0, 40.9, 44.5, 50.4, 55.5, 67.0, 69.0, 84.8, 94.2, 121.2, 126.5, 158.3, 168.0, 170.6, 172.8, 187.5, 200.9. Anal. calcd for C_25_H_31_NO_6_: C, 68.01; H, 7.08; N 3.17. Found: C, 68.09; H, 7.06; N 3.15.

### Biology

#### Antimicrobial activity

*Acinetobacter baumannii* ATCC 17978 and *Staphylococcus aureus* ATCC 29213, well-known as not resistant strains, were evaluated as a Gram-negative and Gram-positive model and were obtained from the American Type Culture Collection. Both strains were grown on blood agar plates (TSA), as described previously.^18^ Minimum inhibitory concentration (MIC) values of steroidal compounds against planktonic bacteria were examined by a broth microdilution method previously described.^20^ Briefly, compounds were dissolved in dimethyl sulfoxide (DMSO) to obtain a concentration of 50 mg mL^−1^. Two-fold serial dilutions ranging from 1 mg mL^−1^ to 2 μg mL^−1^ of the compounds were prepared in triplicate and placed into a polystyrene 96-well plate. Bacterial cell suspensions were prepared at an equivalent to a 0.5 McFarland standard and were subsequently diluted in cation-adjusted Mueller-Hinton broth so that the final culture density was equal to 5 × 106 colony forming unit (cfu) per mL 100 μL of bacteria (5 × 105 cfu) were then added to the microtiter plates containing steroidal compounds. One well with no antibiotic was used as a positive growth control on each plate. The plates were incubated at 37 °C for 18–24 h under shaking (300 rpm) and the MIC was calculated on the basis of concentration of compound in the well having no visible growth. To evaluate the effect of DMSO on bacteria growth kinetics, separate DMSO controls were used. To calculate the minimum bactericidal concentration (MBC), bacterial suspensions from MIC assay microtiter wells were diluted in PBS and spot-plated on TSA plates to count colonies after incubation at 37 °C for 18 h. The MBC was determined as the lowest concentration of substance, which produced ≥99.9% killing (≥3 log 10) after 24 h of incubation as compared to the colony count of the starting inoculum. All tests were performed in triplicate and repeated three times.

#### Hemolysis assay

Cytotoxicity was determined spectrophotometrically by measuring the haemoglobin release from horse erythrocytes.^21^ Briefly, fresh defibrinated horse blood (Oxoid) was centrifuged at 500 × *g* for 5 min and then washed three times with PBS, pH 7.4. Red blood cells were diluted to 4% in PBS and 190 μL were added to 10 μL of 10 (ranging from 512 μg mL^−1^ to 1 mg mL^−1^) in a 96-well plate. After 1 h of incubation at 37 °C, the suspensions were centrifuged for 5 min at 500 × *g*. Then, 150 μL of the supernatant was transferred to a new 96-well plate to measure the absorbance at 450 nm by using a microplate reader, and the percentage of hemolysis was calculated. As a positive control for hemolysis (100% lysis), 1% (v/v) Triton X-100 solution was used. PBS with DMSO (ranging from 1 to 0.0039%) was used as a negative control (0% lysis). Assays were performed in triplicates and repeated twice.

## Conflicts of interest

There are no conflicts to declare.

## Supplementary Material

RA-009-C9RA03673C-s001
