# Supplementary material for: Expeditious synthesis and preliminary antimicrobial activity of deflazacort and its precursors
Source: RSC Adv. 2019 Jul 10;9(37):21519–24. doi: 10.1039/c9ra03673c (PMC9066171; doi:10.1039/c9ra03673c)
Supplement: RA-009-C9RA03673C-s001 [file RA-009-C9RA03673C-s001.pdf]

Electronic Supplementary Material (ESI) for RSC Advances.
This journal is © The Royal Society of Chemistry 2019

SUPPORTING INFORMATION

Expeditious Synthesis and Preliminary Antimicrobial Activity of Deflazacort and its Precursors

Anna Esposito,[a] Eliana De Gregorio,[b] Maria De Fenza,[a] Daniele D'Alonzo,[a] Anil Satawani,[c] and Annalisa Guaragna[a]*

[a]Department of Chemical Sciences, University of Napoli Federico II, via Cintia, 80126 Napoli, (Italy); [b]Department of Molecular Medicine and Medical Biotechnologies, via S. Pansini, 5, 80131 Napoli (Italy); Symbiotec Pharmalab pvt ltd, Pithampur, Indore (India).

annalisa.guaragna@unina.it (A.G.)

TABLE OF CONTENTS

CHEMICAL SYNTHESIS S2

COPIES OF NMR SPECTRA S6

LC-MS DATA S17

BIOLOGICAL ASSAYS S20

REFERENCES S21

CHEMICAL SYNTHESIS

General methods and materials.

All chemicals and solvents were purchased with the highest degree of purity (Sigma-Aldrich, Alfa Aesar, VWR) and used without further purification. 9-Bromotriene acetate **7** was provided by Symbiotec Pharmalab PVT. All moisture-sensitive reactions were performed under nitrogen atmosphere using oven-dried glassware. The reactions were monitored by TLC (precoated silica gel plate F254, Merck) and the products were detected by exposure to ultraviolet radiation, iodine vapor, and chromic mixture. Column chromatography: Merck Kieselgel 60 (70-230 mesh); flash chromatography: Merck Kieselgel 60 (230-400 mesh). The purity of the synthetic intermediates and the final compound was determined by CHNS analysis and was ≥ 95% in all cases. NMR spectra were recorded on NMR spectrometers operating at 400 MHz (Bruker DRX, Bruker AVANCE) or 500 MHz (Varian Inova), using $CDCl_3$ solutions unless otherwise specified. Coupling constant values (*J*) were reported in Hz.

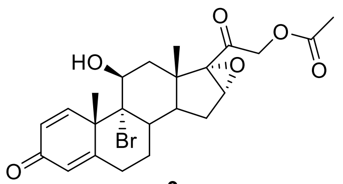

9

1,4-Pregnadiene-9-bromo-11-hydroxy-16α,17α-epoxy-3,20-dione (9). To a stirred solution of 9-bromotriene acetate **7** (1.0 g, 2.16 mmol) in anhydrous CH_2Cl_2 (30 mL), *m*-CPBA (0.75 g, 4.32 mmol) was added at room temperature. The mixture was warmed to reflux and stirred for 16 h. Then, aq. $NaHCO_3$ was added and the mixture was extracted with CH_2Cl_2; the organic layer was dried (Na_2SO_4) and the solvent evaporated under reduced pressure. The solid residue was recrystallized from AcOEt to give the final compound **9** (0.49 g, 47% yield) as a white solid. ^{1}H NMR (400 MHz): δ 1.41 (s, 3H), 1.48-1.55 (m, 1H), 1.70 (s, 3H), 1.71-1.82 (m, 2H), 1.94-2.05 (m, 3H), 2.15 (s, 3H), 2.16–2.25 (m, 1H), 2.36–2.49 (m, 2H), 2.55–2.68 (m, 1H), 3.85 (s, 1H), 4.59 (d, *J* = 13.4, 1H), 4.67 (d, *J* = 13.4, 1H), 4.76 (bs, 1H), 6.07 (bs, 1H), 6.32 (d, *J* = 10.1, 1H), 7.21 (d, *J* = 10.1, 1H). ^{13}C NMR (100 MHz): 18.3, 20.4, 24.9, 27.0, 28.3, 30.4, 33.5, 37.3, 39.3, 42.1, 50.2, 61.1, 65.7, 70.5, 75.9, 85.3, 125.1, 129.3, 152.3, 165.5, 170.4, 186.2, 198.9. Anal. calcd for $C_{23}H_{27}BrO_6$: C, 57.63; H, 5.68; Br, 16.67. Found: C, 57.75; H, 5.66; Br, 16.62.

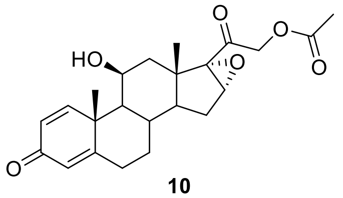

10

1,4-Pregnadiene-11-hydroxy-16α,17α-epoxy-3,20-dione (10). To a boiling and stirring suspension of epoxide **9** (0.50 g, 1.04 mmol) in anhydrous THF (13.0 mL), a solution of Bu_3SnH (0.36 mL, 1.25

mmol) and AIBN (catalytic amount, 33.3 mg, 0.21 mmol) in THF (10 mL) was added dropwise under nitrogen atmosphere. The reaction mixture was stirred for 30 minutes at reflux temperature. The crude residue was then concentrated under reduced pressure and recrystallized from AcOEt to give the final compound **10** (0.41 g, 98% yield). Epoxide **10** (40% yield) was also obtained from compound **8** by epoxidation reaction using the same procedure reported for **9**. Data for **10**: white solid, ^{1}H NMR (400 MHz): δ 1.02-1.14 (m, 2H), 1.39 (s, 3H), 1.46 (s, 3H), 1.46-1.51 (m, 2H), 1.62-1.70 (m, 1H), 1.97-2.02 (m, 3H), 2.15 (s, 3H), 2.15–2.19 (m, 1H), 2.29–2.38 (m, 1H), 2.51–2.62 (m, 1H), 3.82 (s, 1H), 4.42 (bs, 1H), 4.57 (d, *J* = 13.4, 1H), 4.66 (d, *J* = 13.4, 1H), 6.00 (bs, 1H), 6.26 (dd, *J* = 1.8, 10.1, 1H), 7.21 (d, *J* = 10.1, 1H). ^{13}C NMR (100 MHz): 17.4, 20.5, 21.3, 27.8, 28.3, 29.5, 31.9, 33.4, 41.0, 42.0, 44.2, 45.1, 56.2, 61.4, 65.8, 69.9, 122.6, 128.0, 156.1, 169.6, 170.4, 186.6, 190.0. Anal. calcd for $C_{23}H_{28}O_6$: C, 68.98; H, 7.05. Found: C, 69.05; H, 7.03.

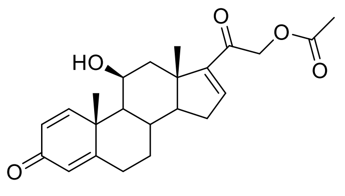

8

1,4,17-Pregnatriene-11-hydroxy-3,20-dione (**8**). Compound **8** was obtained in 93% yield from **7** by using the same conditions reported above for preparation of **10**. Data for **8**: white solid, ^{1}H NMR (500 MHz): δ 1.06-1.21 (m, 1H), 1.26 (s, 3H), 1.32-1.38 (m, 1H), 1.49 (s, 3H), 1.59-1.63 (m, 2H), 2.06-2.12 (m, 1H), 2.17 (s, 3H), 2.18-2.30 (m, 2H), 2.33–2.43 (m, 2H), 2.45–2.51 (m, 1H), 2.55–2.67 (m, 1H), 4.40 (bs, 1H), 4.85 (d, *J* = 16.1, 1H), 5.01 (d, *J* = 16.1, 1H), 6.01 (bs, 1H), 6.27 (d, *J* = 10.1, 1H), 6.73 (bs, 1H), 7.31 (d, *J* = 10.1, 1H). ^{13}C NMR (100 MHz): 18.4, 20.5, 21.2, 30.1, 31.8, 32.8, 33.6, 44.2, 44.6, 46.1, 56.1, 56.4, 65.5, 70.2, 122.5, 128.0, 143.5, 152.2, 156.1, 169.4, 170.2, 186.6, 190.5. Anal. calcd for $C_{23}H_{28}O_5$: C, 71.85; H, 7.34. Found: C, 71.80; H, 7.35.

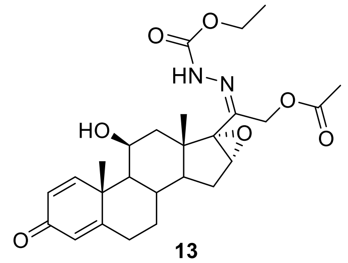

13

20-Carboethoxyhydrazone of 1,4-pregnadiene-11-hydroxy-16α,17α-epoxy-3-one (**13**). To a stirring suspension of **10** (0.50 g, 1.25 mmol) in anhydrous 1,4-dioxane (24 mL), ethyl carbazate (0.26 g, 2.5 mmol) and *p*-toluenesulfonic acid (0.24 g, 1.25 mmol) were sequentially added at room temperature under argon atmosphere. The resulting mixture was stirred at the same temperature for 8 h. Then aq $NaHCO_3$ was added and the mixture was extracted with EtOAc; the organic layer was dried (Na_2SO_4) and the solvent evaporated under reduced pressure. The solid residue was recrystallized from Et_2O to give the final compound **13** (0.36 g, 59 % yield) as a white solid. ^{1}H NMR (500 MHz): δ 1.01-1.12 (m, 2H), 1.15-1.27 (m, 2H), 1.31 (s, 3H), 1.33 (t, *J* = 7.1, 3H),

1.40-1.44 (m, 1H), 1.47 (s, 3H), 1.68 (dd, *J* = 3.1, 14.2, 1H), 1.96-2.04 (m, 2H), 2.13 (s, 3H), 2.15-2.18 (m, 1H), 2.32 (dd, *J* = 3.0, 12.7, 1H), 2.50–2.61 (m, 2H), 3.67 (s, 1H), 4.22-4.29 (m, 2H), 4.31 (d, *J* = 13.1, 1H), 4.40 (bs, 1H), 4.56 (d, *J* = 13.1, 1H), 6.00 (bs, 1H), 6.25 (d, *J* = 10.1, 1H), 7.29 (d, *J* = 10.1, 1H). ^{13}C NMR (125 MHz): 14.6, 18.2, 20.6, 21.2, 27.2, 29.9, 31.9, 33.5, 41.4, 41.7, 44.2, 45.9, 55.0, 56.2, 59.6, 62.1, 69.8, 70.2, 122.5, 127.8, 142.5, 153.6, 156.2, 169.7, 171.2, 186.6. Anal. calcd for $C_{26}H_{34}N_2O_7$: C, 64.18; H, 7.04; N 5.76. Found: C, 64.29; H, 7.02; N 5.75.

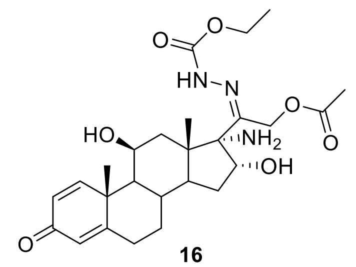

16

20-Carboethoxyhydrazone of 1,4-pregnadiene-16α-amino-11,17α-diol-3-one (**16**). *Method A.* Epoxide **13** (0.30 g, 0.62 mmol) was dissolved in anhydrous 1,4-dioxane (15 mL) under nitrogen atmosphere at rt. Anhydrous ammonia was then gently bubbled into the solution for 3 minutes and the reaction was stirred at the same temperature for 18 h. Nitrogen was bubbled in the reaction mixture until the ammonia was eliminated from the solution (pH = 7). The mixture was diluted with CH_2Cl_2 and the organic layers were washed with brine, dried (Na_2SO_4) and concentrated under reduced pressure. The solid residue was recrystallized from Et_2O to give the final compound **16** (0.30 g, 97 % yield) as a white solid. *Method B (one-pot procedure)*. To a stirred suspension of **10** (0.50 g, 1.25 mmol) in anhydrous 1,4-dioxane (24 mL), ethyl carbazate (0.26 g, 2.5 mmol) and *p*-toluenesulfonic acid (0.24 g, 1.25 mmol) were sequentially added at room temperature under argon atmosphere. The resulting mixture was stirred at the same temperature for 8 h and then anhydrous ammonia was gently bubbled into the solution for 3 minutes. The reaction mixture was stirred at room temperature for 18h. The mixture was diluted with CH_2Cl_2 and the organic layers were washed with brine, dried (Na_2SO_4) and concentrated under reduced pressure. The solid residue was recrystallized from Et_2O to give the final compound **16** (0.43 g, 69 % overall yield). ^1H NMR (400 MHz, acetone-d_6): δ 1.04-1.16 (m, 5H), 1.21-1.41 (m, 4H), 1.51 (s, 3H), 1.52-1.57 (m, 1H), 1.78-1.99 (m, 4H), 2.03-2.17 (m, 7H), 2.35 (dd, *J* = 3.4, 13.4, 1H), 2.62 (td, *J* = 4.9, 13.4, 1H), 3.81 (bs, 1H), 4.17 (q, *J* = 7.1, 2H), 4.47 (bs, 1H), 4.76 (d, *J* = 13.0, 1H), 4.90 (d, *J* = 13.0, 1H), 5.16 (dd, *J* = 3.0, 9.2, 1H), 5.92 (bs, 1H), 6.14 (dd, *J* = 1.6, 10.1, 1H), 7.33 (d, *J* = 10.1, 1H). ^{13}C NMR (100 MHz, acetone-d_6): 14.0, 18.2, 19.9, 20.8, 29.7, 30.9, 31.6, 33.9, 40.9, 44.0, 46.4, 48.9, 55.6, 58.2, 60.8, 69.4, 81.2, 83.7, 121.8, 127.3, 149.4, 153.6, 155.9, 170.1, 170.3, 185.1. Anal. calcd for $C_{26}H_{37}N_3O_7$: C, 62.01; H, 7.41; N 8.34. Found: C, 62.08; H, 7.38; N 8.36.

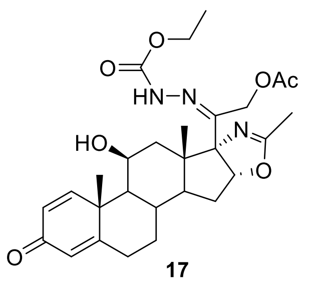

17

20-Carboethoxyhydrazone of 1,4-pregnadiene-11-hydroxy-16α,17α-oxazole-3-one (**17**). I_2 (0.30 g, 1.19 mmol) was added to a stirred solution of polymer supported triphenylphosphine (PS-TPP; 100-200 mesh, extent of labeling: ~3 mmol/g triphenylphosphine loading) (0.40 g, 1.19 mmol) in anhydrous DCM (20 mL) at rt. Then glacial acetic acid (35 μL, 0.59 mmol) was added and the solution was stirred at room temperature for 20'. Afterwards, **16** (0.30 g, 0.59 mmol) and imidazole (0.16 g, 2.38 mmol) were sequentially added and the solution was warmed to 40 °C and stirred for 2 h. The mixture was then filtered and the solvent removed under reduced pressure. The solid residue was recrystallized from Et_2O to give the final compound **17** (0.29 g, 96% yield) as a white solid. ^{1}H NMR (500 MHz, acetone-d_6): δ 1.04 (dd, *J* = 3.7, 11.4, 1H), 1.07 (s, 1H), 1.09-1.18 (m, 1H), 1.27 (t, *J* = 7.1, 1H), 1.29-1.36 (m, 3H), 1.51 (s, 3H), 1.74 (dd, *J* = 5.7, 13.6, 1H), 1.81 (dd, *J* = 7.6, 13.6, 1H), 1.85-1.90 (m, 4H), 2.12 (s, 3H), 2.18-2.25 (m, 1H), 2.35 (dd, *J* = 3.4, 13.5, 1H), 2.65 (td, *J* = 5.7, 13.5, 1H), 3.83 (bs, 1H), 4.17 (q, *J* = 7.1, 2H), 4.47 (bs, 1H), 4.73 (d, *J* = 13.0, 1H), 4.92 (d, *J* = 13.0, 1H), 5.66 (d, *J* = 5.7, 1H), 5.92 (bs, 1H), 6.14 (dd, *J* = 1.8, 10.1, 1H), 7.31 (d, *J* = 10.1, 1H), 9.37 (bs, 1H). ^{13}C NMR (125 MHz, acetone-d_6): 13.2, 14.0, 19.1, 19.9, 20.8, 29.7, 30.6, 31.5, 33.9, 41.7, 43.9, 47.0, 50.4, 55.5, 57.5, 60.9, 69.1, 83.7, 91.6, 121.9, 127.4, 145.9, 153.4, 155.7, 165.1, 169.5, 170.2, 185.0. Anal. calcd for $C_{28}H_{37}N_3O_7$: C, 63.74; H, 7.07; N 7.96. Found: C, 63.81; H, 7.02; N 7.99.

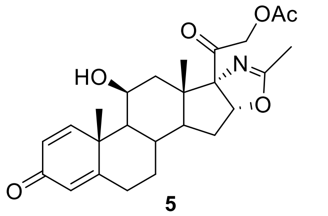

5

Deflazacort (**5**). To a stirred solution of **17** (0.30 g, 0.57 mmol) in acetone (8 mL), HCl 37% solution (0.09 mL, 1.14 mmol) was added and the solution was stirred at rt for 24h. Then aq. $NaHCO_3$ was added and the mixture was extracted with CH_2Cl_2; the combined organic layers were washed with brine, dried (Na_2SO_4) and the solvent was evaporated under reduced pressure. Chromatography of the crude residue over silica gel (hexane:acetone = 7:3) gave the pure DFZ (**5**) (0.18 g, 72% yield) as a white solid. ^{1}H NMR (400 MHz, CD_3OD): δ 1.01 (dd, *J* = 3.6, 11.3, 1H), 1.02 (s, 3H), 1.06-1.25 (m, 2H), 1.49 (s, 3H), 1.75 (dd, *J* = 5.9, 13.8, 1H), 1.79-1.85 (m, 1H), 1.89 (dd, *J* = 3.9, 14.2, 1H), 1.97 (s, 3H), 2.01 (dd, *J* = 2.7, 13.8, 1H), 2.06-2.12 (m, 1H), 2.13 (s, 3H), 2.20 (dd, *J* = 4.1, 11.6, 1H), 2.37 (ddd, *J* = 1.8, 4.6, 13.4, 1H), 2.65 (td, *J* = 5.7, 13.4, 1H), 4.41 (dd, *J* = 3.2, 6.3, 1H), 4.93 (s, 2H), 5.30 (d, *J* = 5.5, 1H), 6.00 (bs, 1H), 6.25 (dd, *J* = 1.9, 10.1, 1H), 7.45 (d, *J* = 10.1, 1H). ^{13}C NMR (125 MHz, CD_3OD): 12.5, 17.2, 18.9, 20.1, 30.4, 31.6, 33.9, 34.0, 40.9, 44.5, 50.4, 55.5, 67.0, 69.0, 84.8, 94.2, 121.2, 126.5, 158.3, 168.0,

170.6, 172.8, 187.5, 200.9. Anal. calcd for $C_{25}H_{31}NO_6$: C, 68.01; H, 7.08; N 3.17. Found: C, 68.09; H, 7.06; N 3.15.

COPIES OF ^{1}H, ^{13}C AND ^{1}H-^{1}H COSY NMR SPECTRA

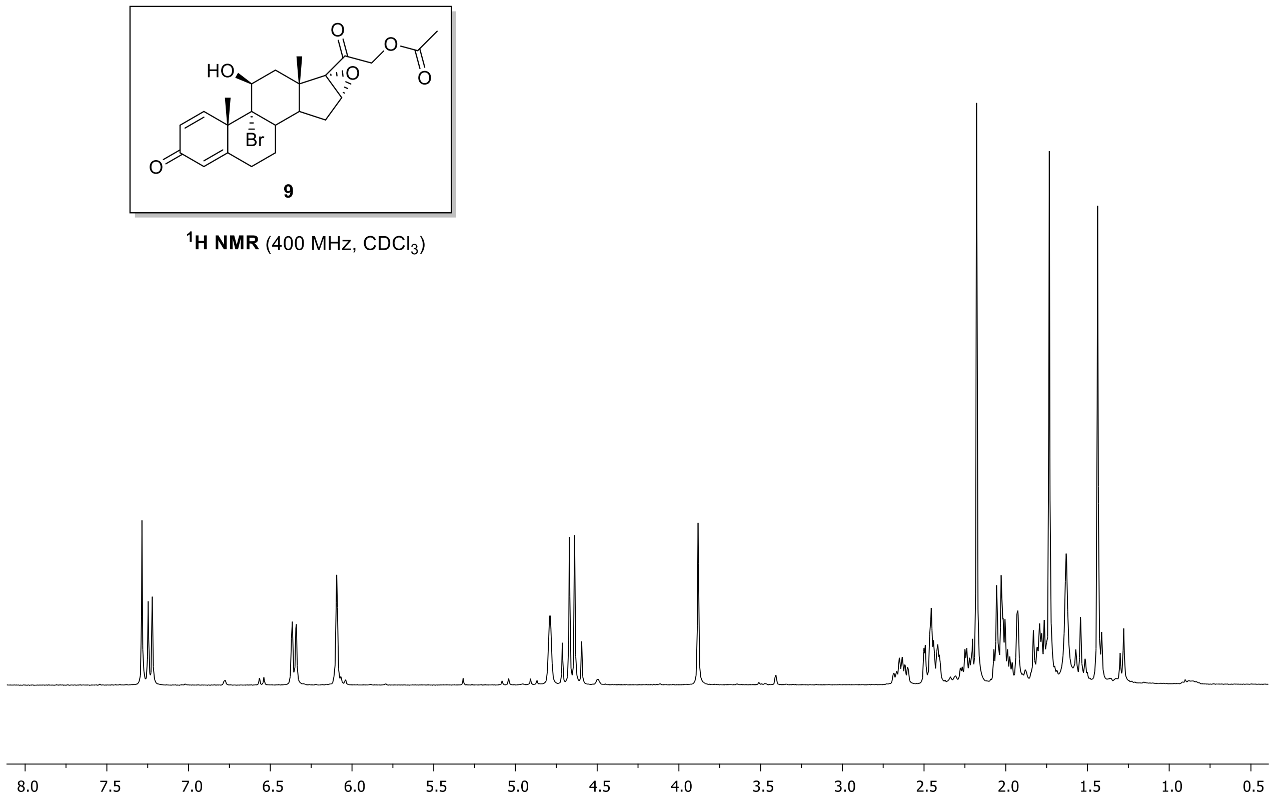

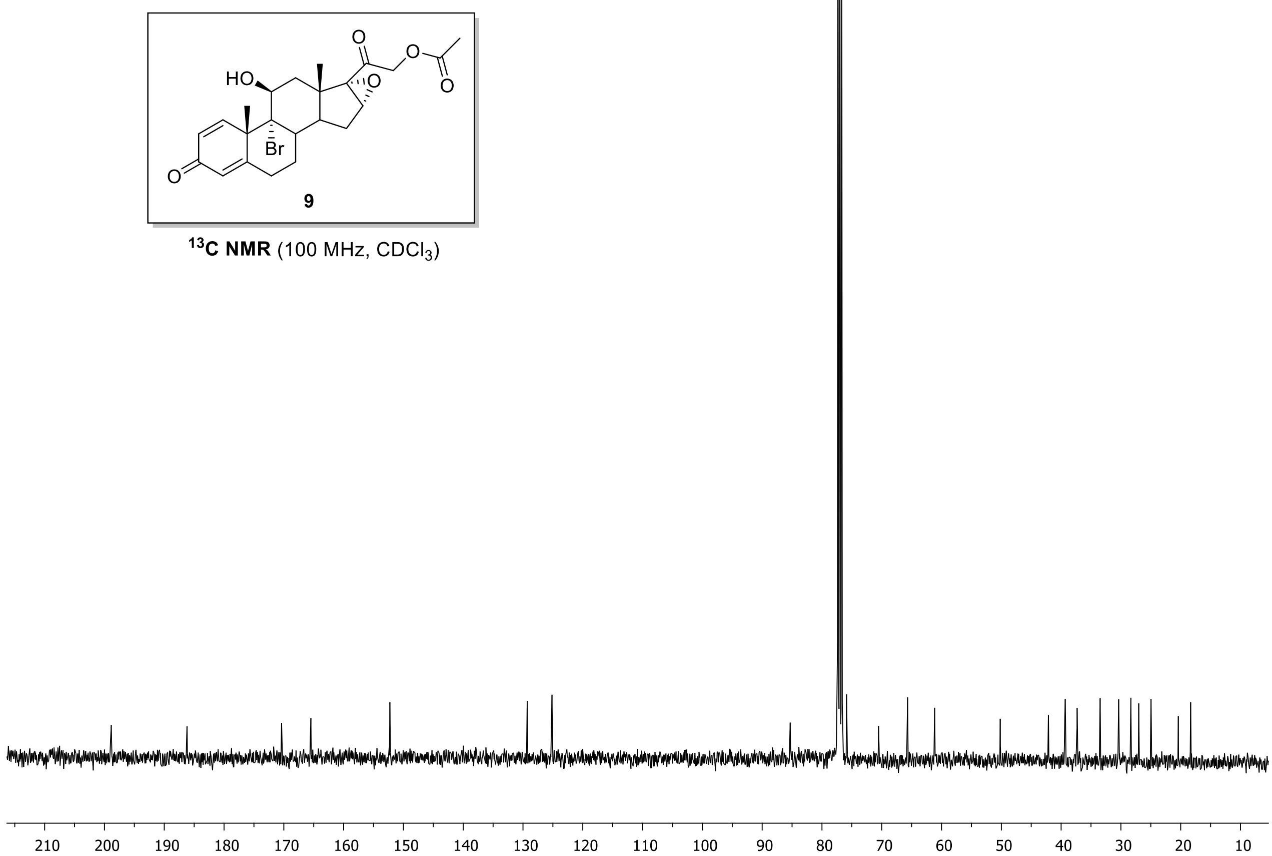

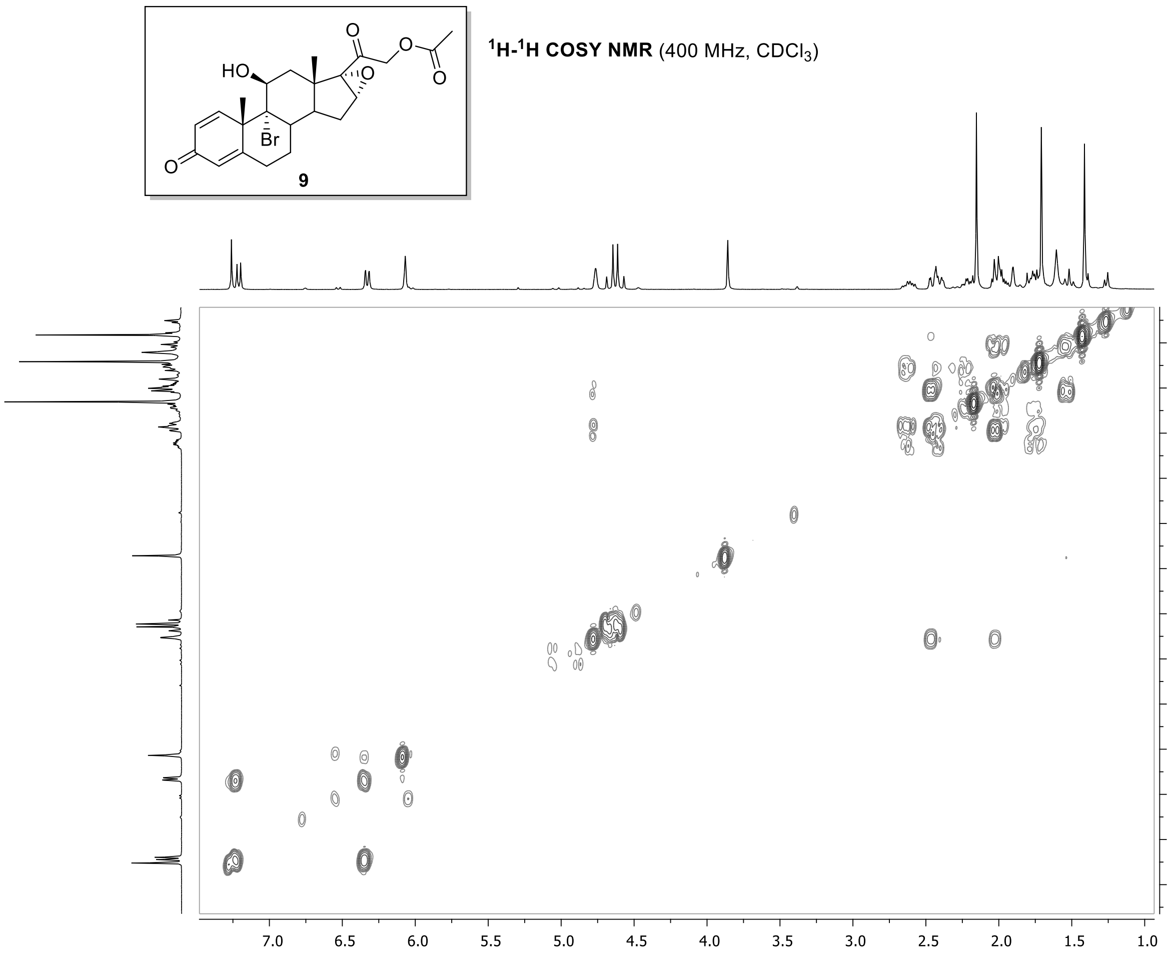

^{1}H-^{1}H COSY NMR (400 MHz, $CDCl_3$)

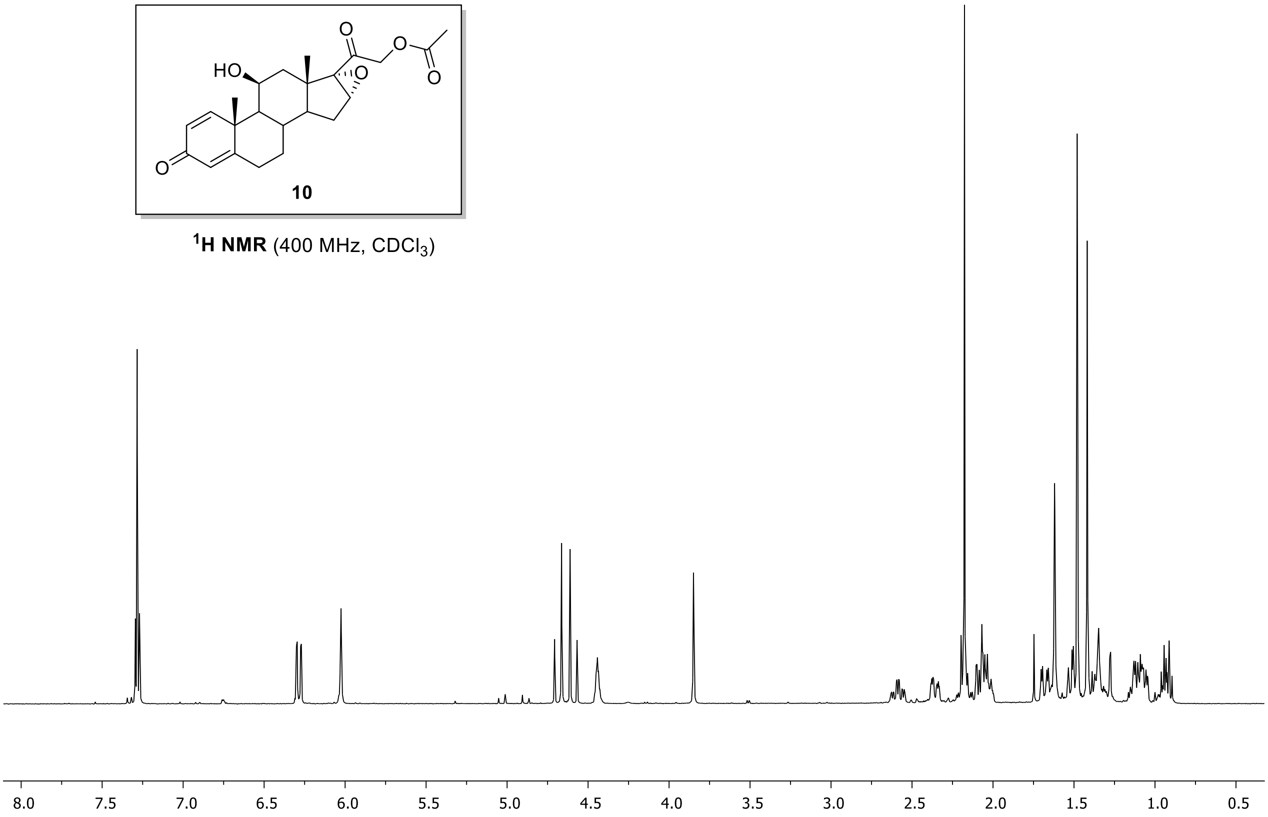

^{1}H NMR (400 MHz, $CDCl_3$)

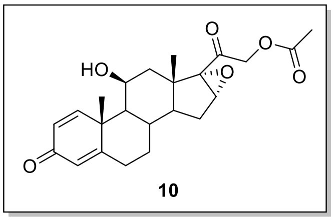

^{13}C NMR (100 MHz, $CDCl_3$)

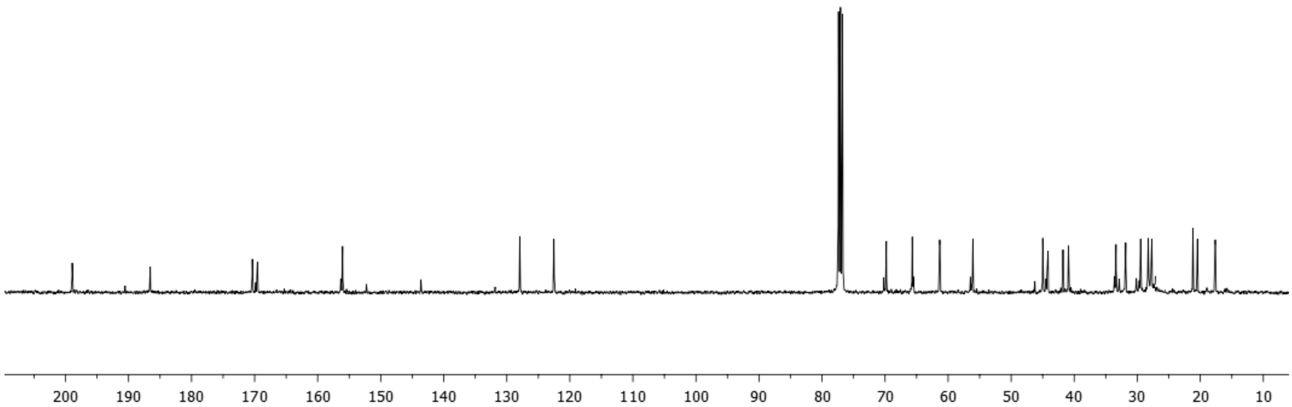

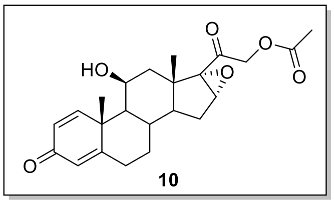

^{1}H-^{1}H COSY NMR (400 MHz, $CDCl_3$)

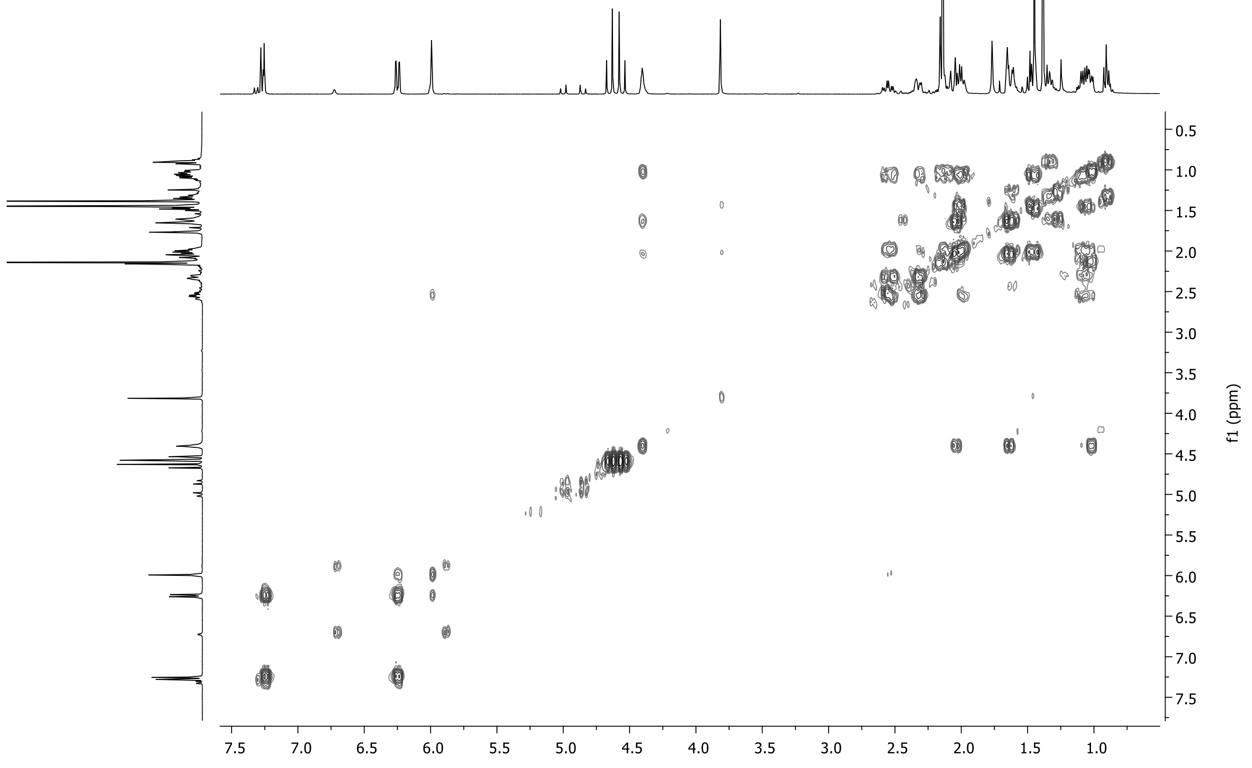

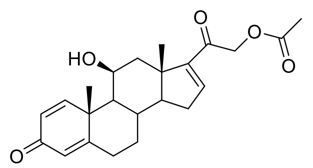

8

^{1}H NMR (500 MHz, $CDCl_3$)

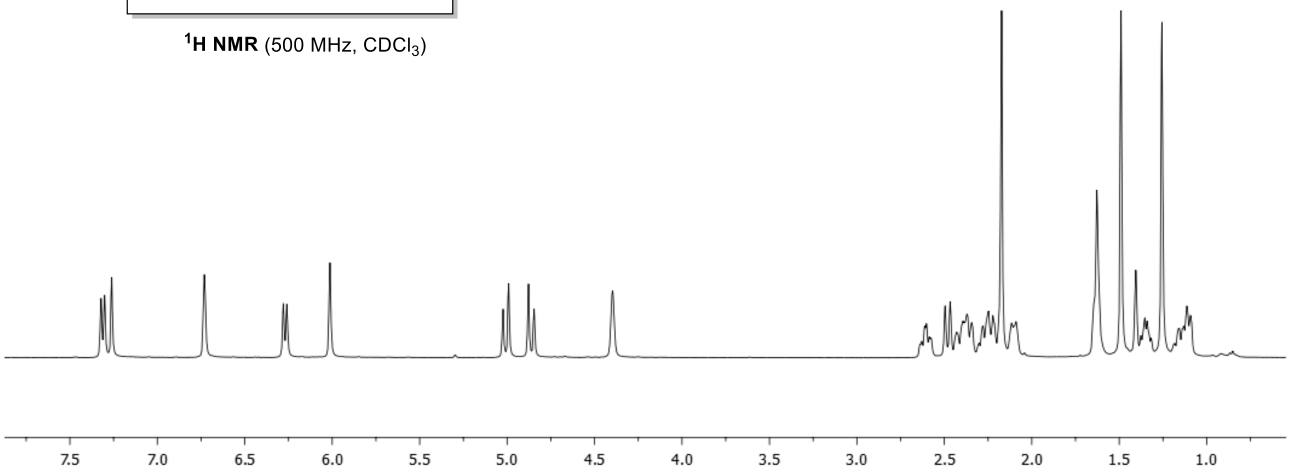

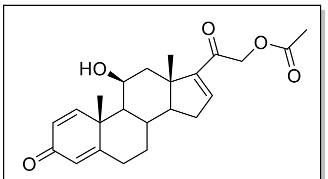

8

^{13}C NMR (125 MHz, $CDCl_3$)

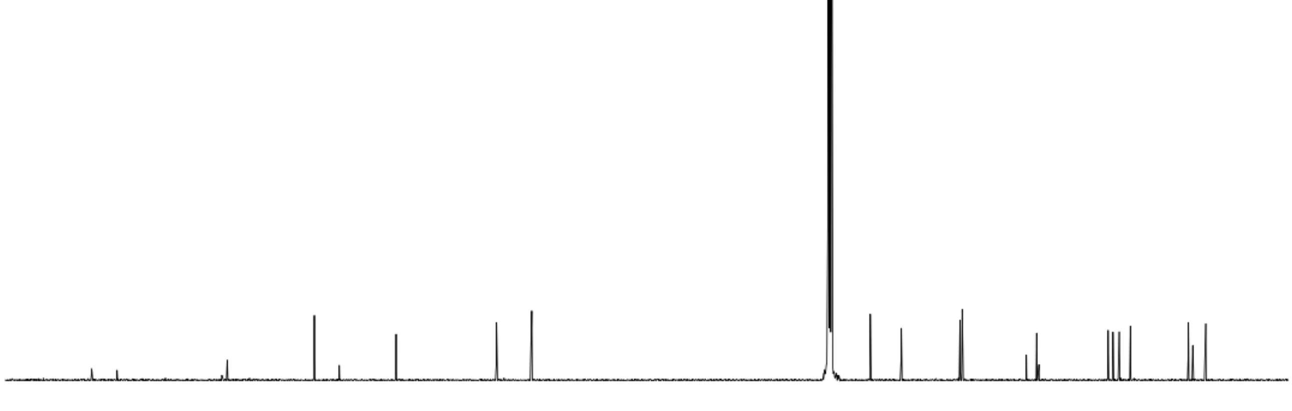

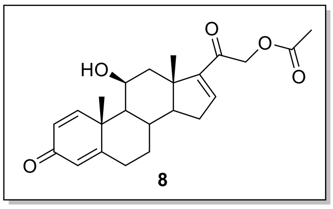

^{1}H-^{1}H COSY NMR (500 MHz, $CDCl_3$)

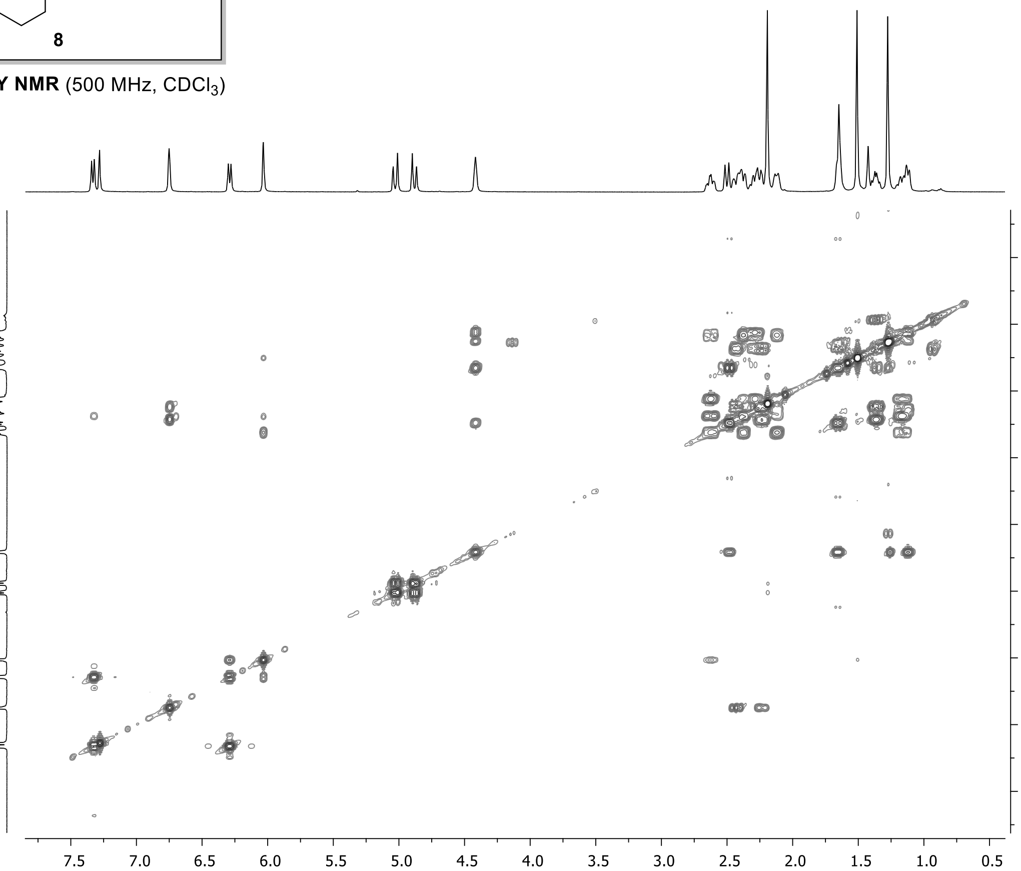

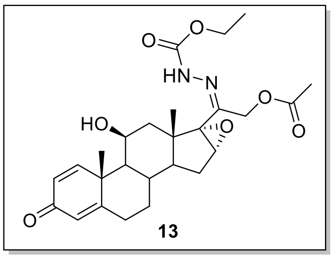

^{1}H NMR (500 MHz, $CDCl_3$)

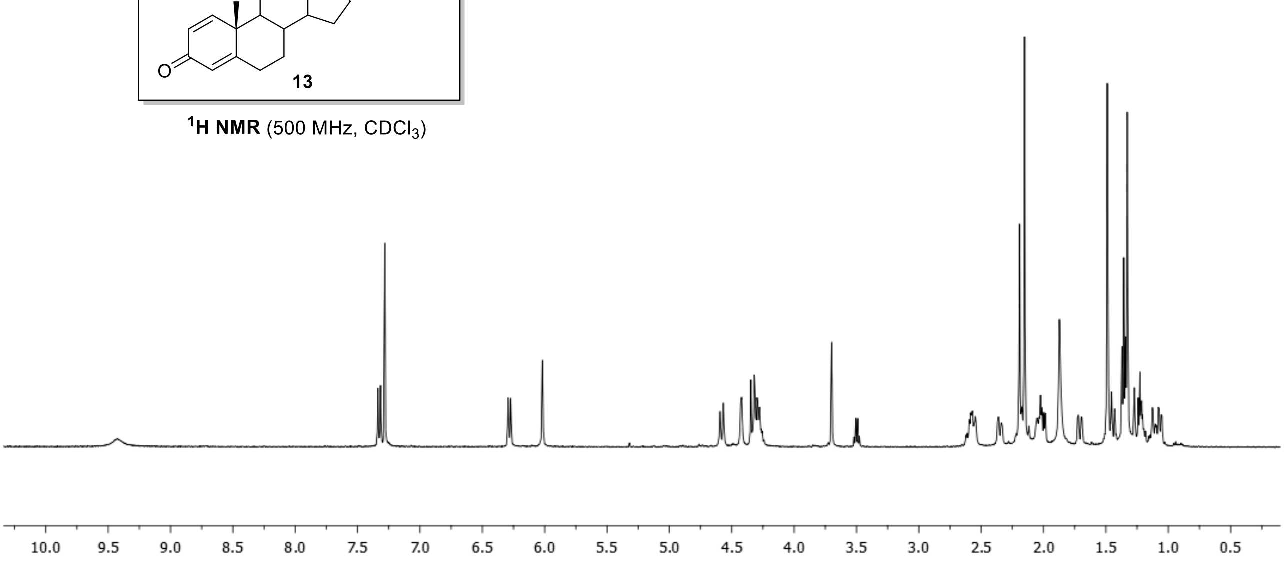

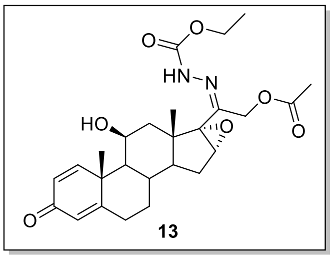

^{13}**C NMR** (125 MHz, $CDCl_3$)

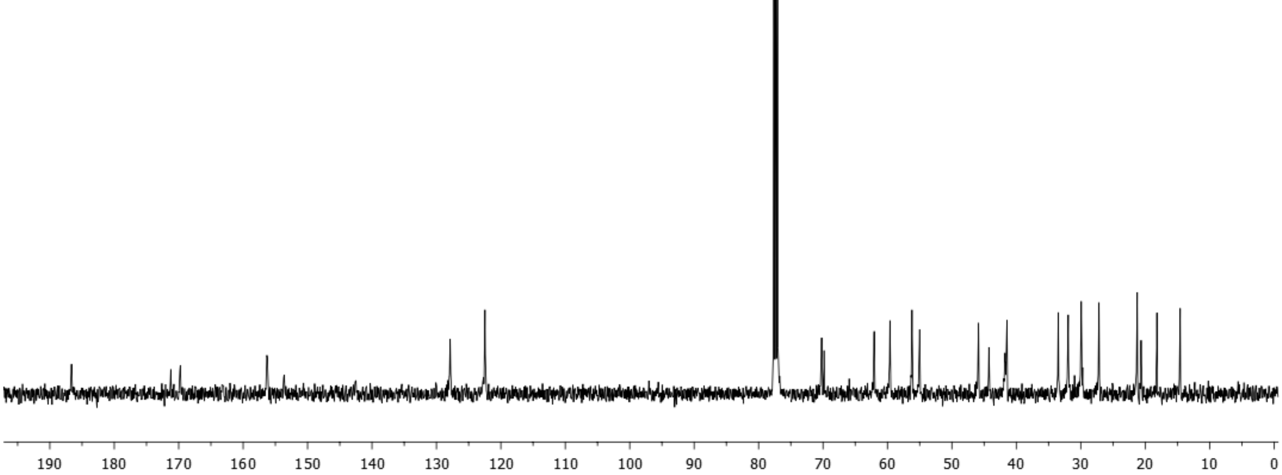

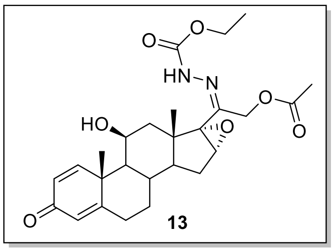

^{1}**H-**^{1}**H COSY NMR** (500 MHz, $CDCl_3$)

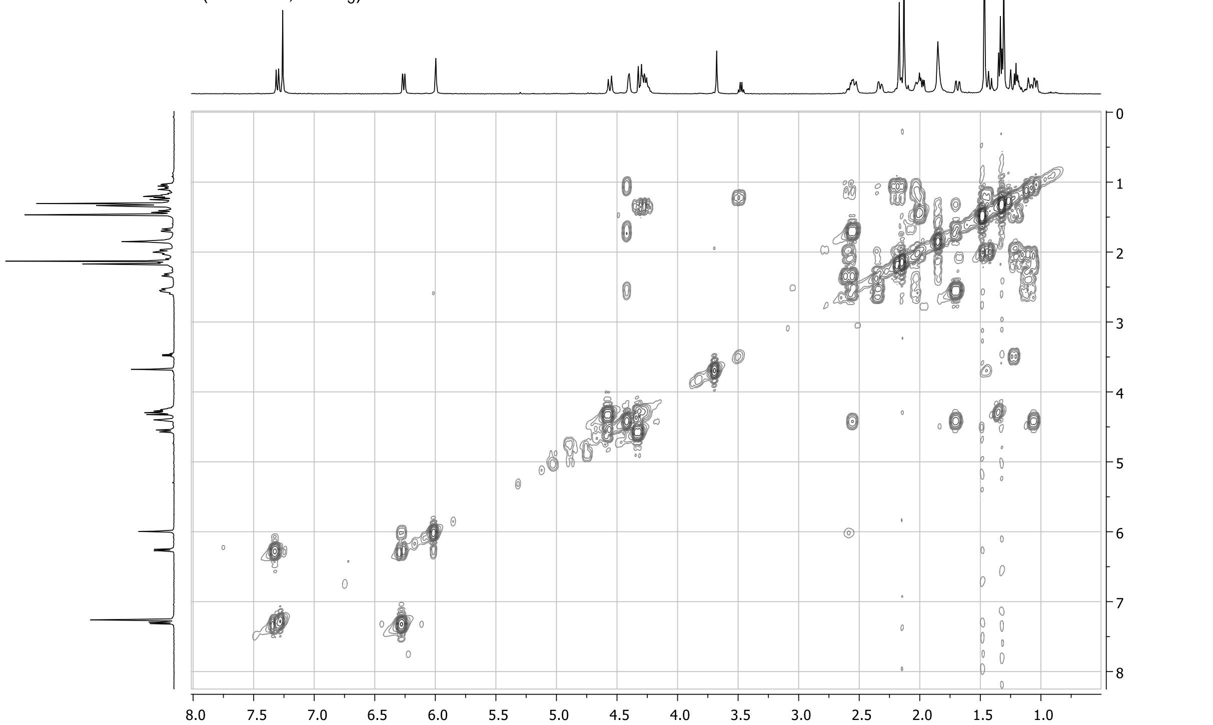

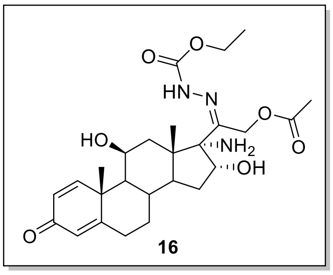

^{1}**H NMR** (400 MHz, acetone-d_6)

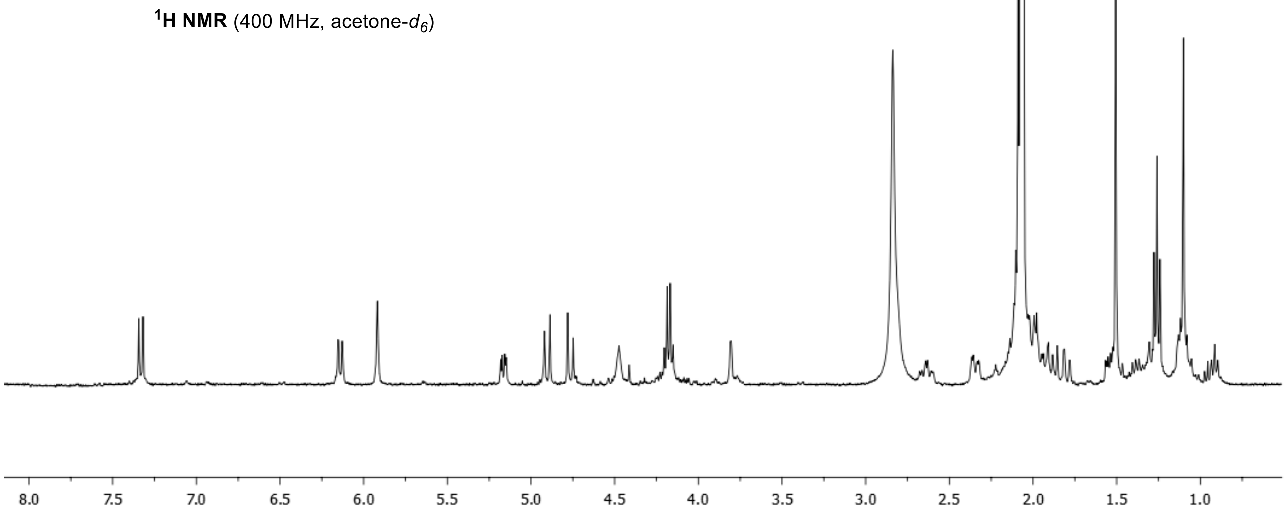

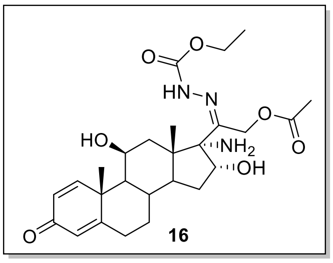

^{1}**H NMR** (400 MHz, acetone-d_6)

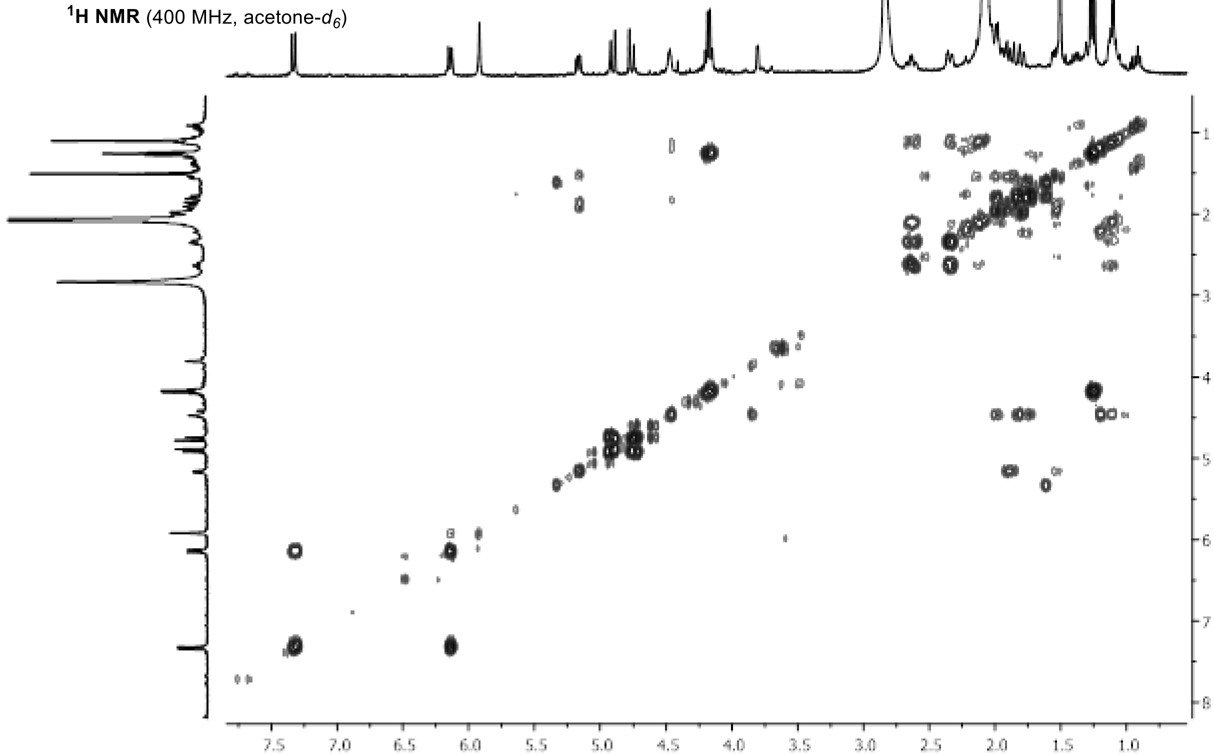

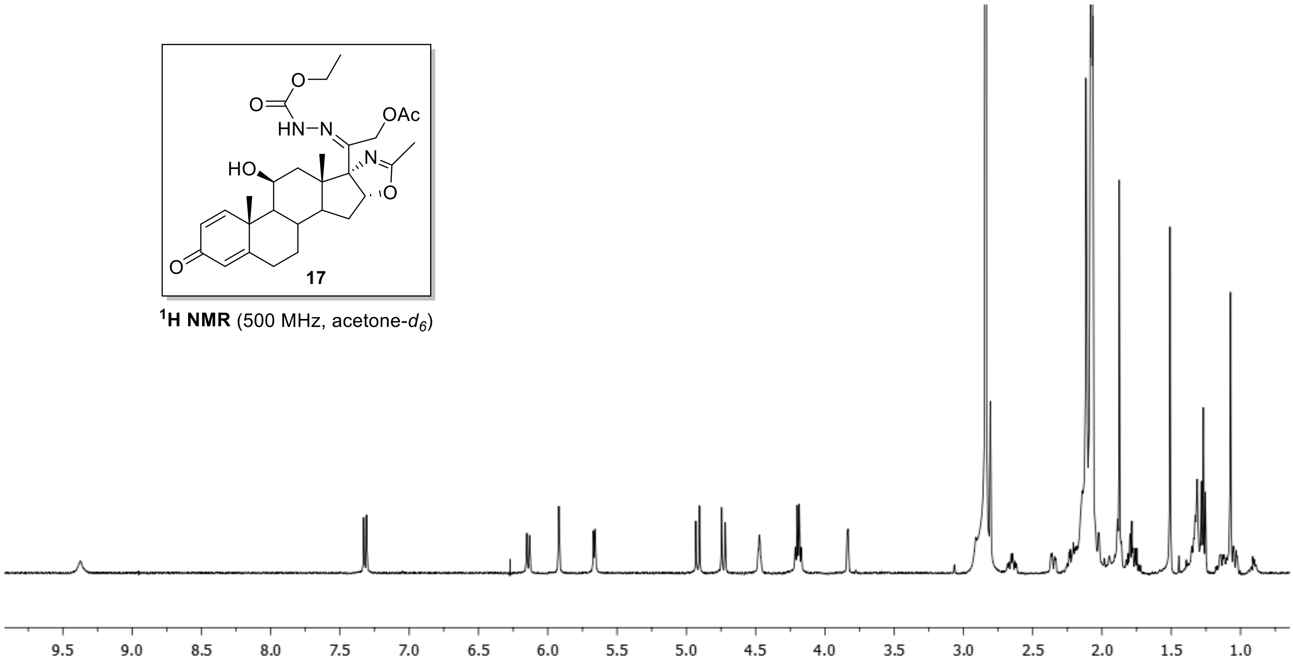

^{1}H NMR (500 MHz, acetone-d_6)

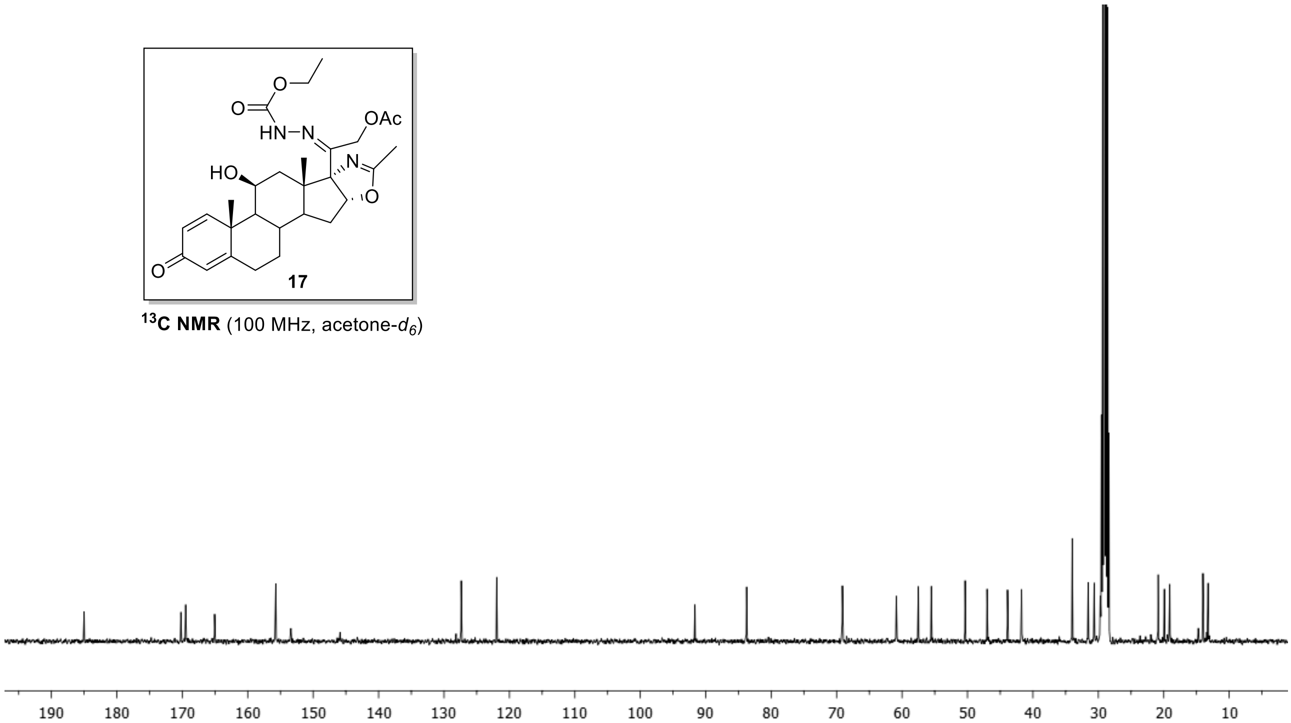

^{13}C NMR (100 MHz, acetone-d_6)

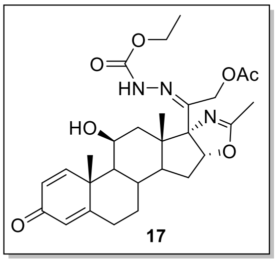

^{1}H-^{1}H COSY NMR (400 MHz, acetone-d_6)

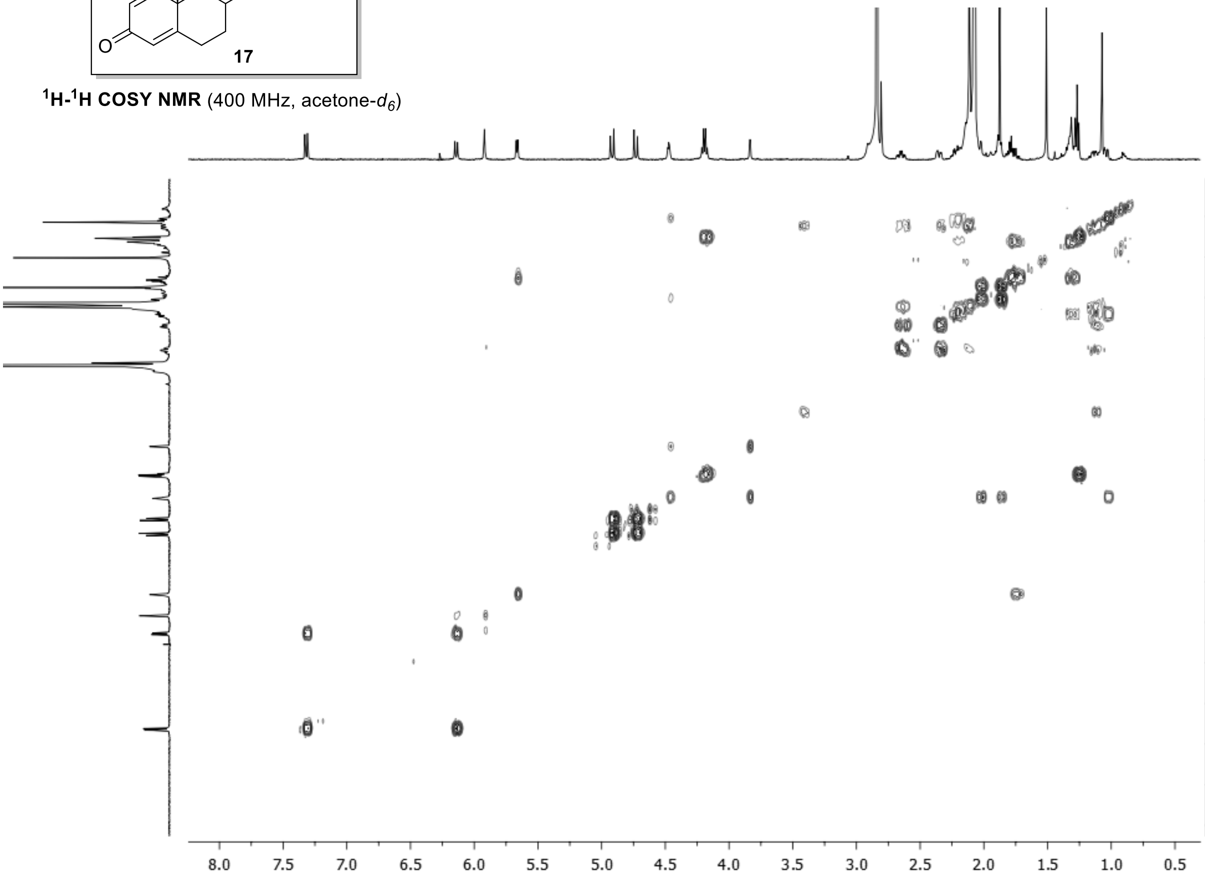

^{1}H NMR (400 MHz, CD_3OD)

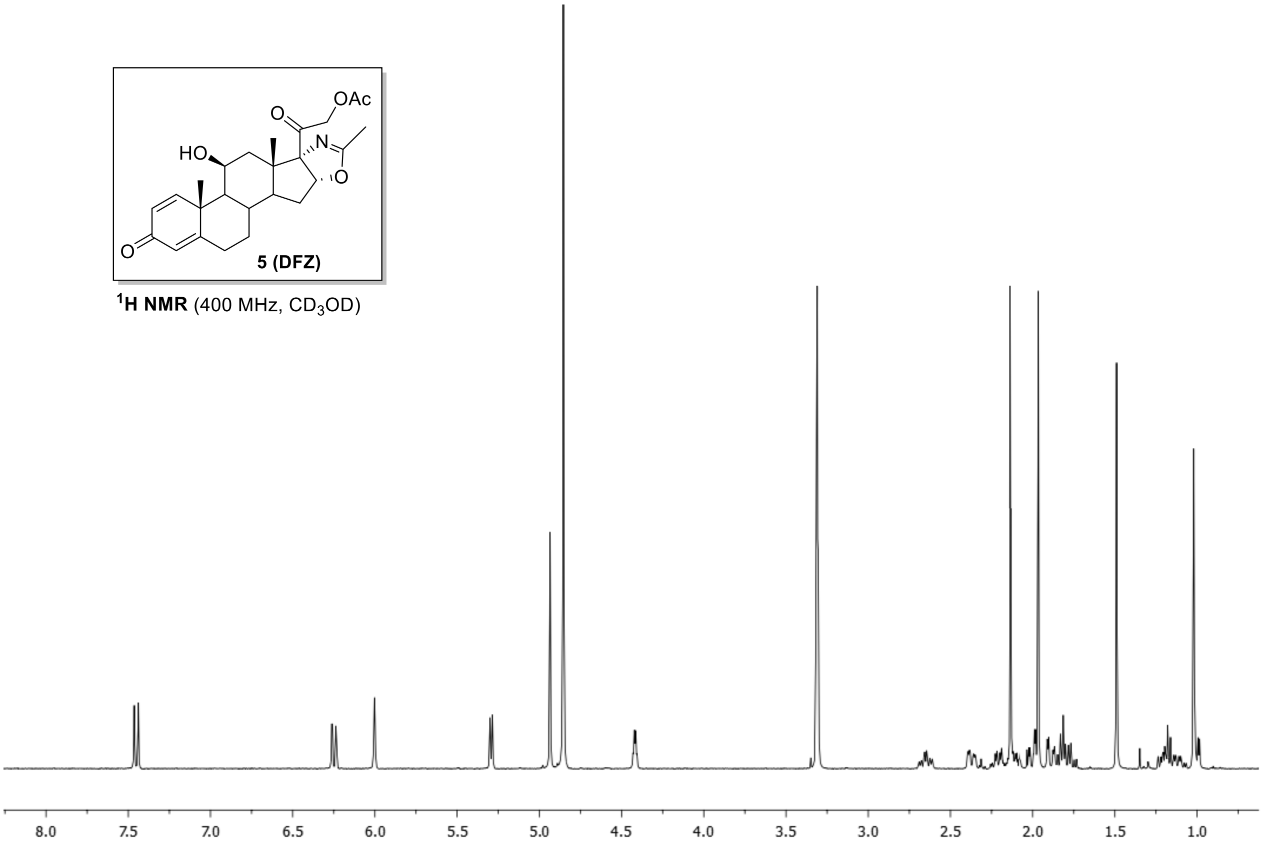

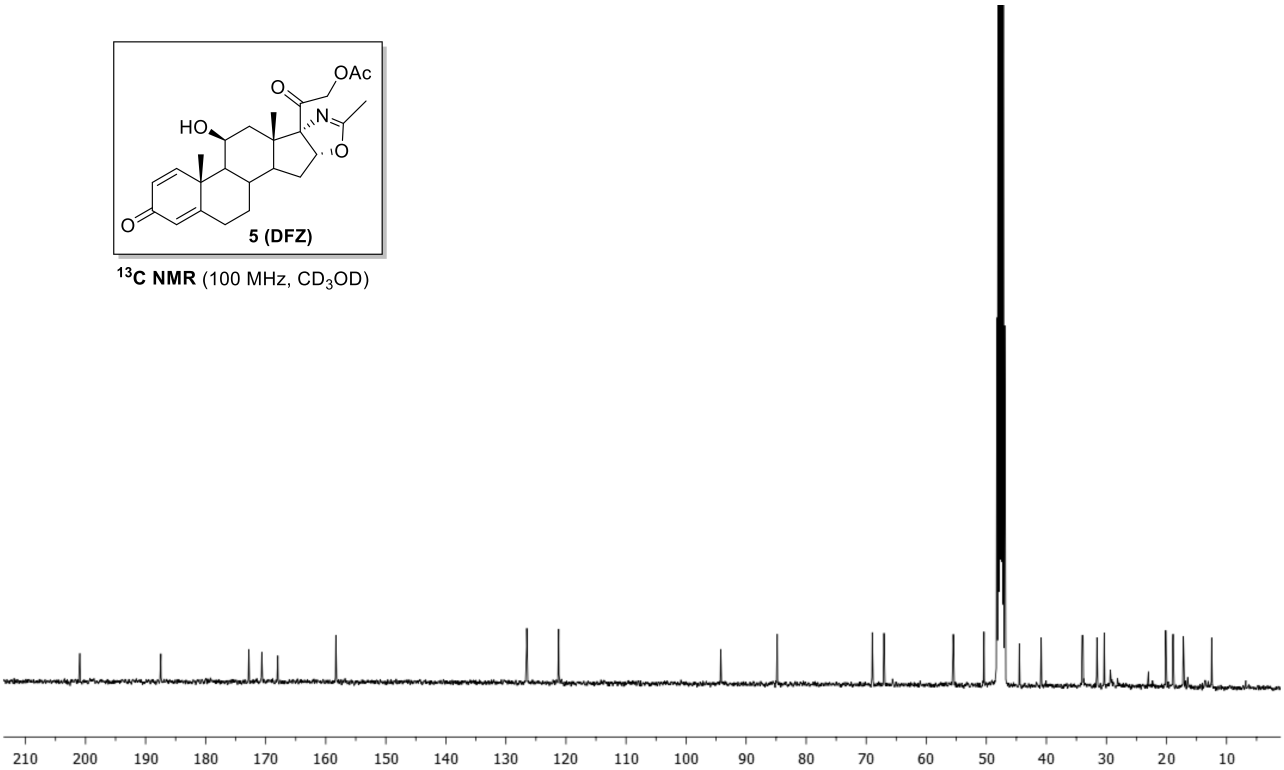

^{13}C NMR (100 MHz, CD_3OD)

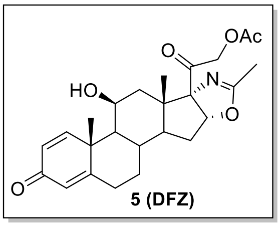

^{1}H-^{1}H COSY NMR (400 MHz, CD_3OD)

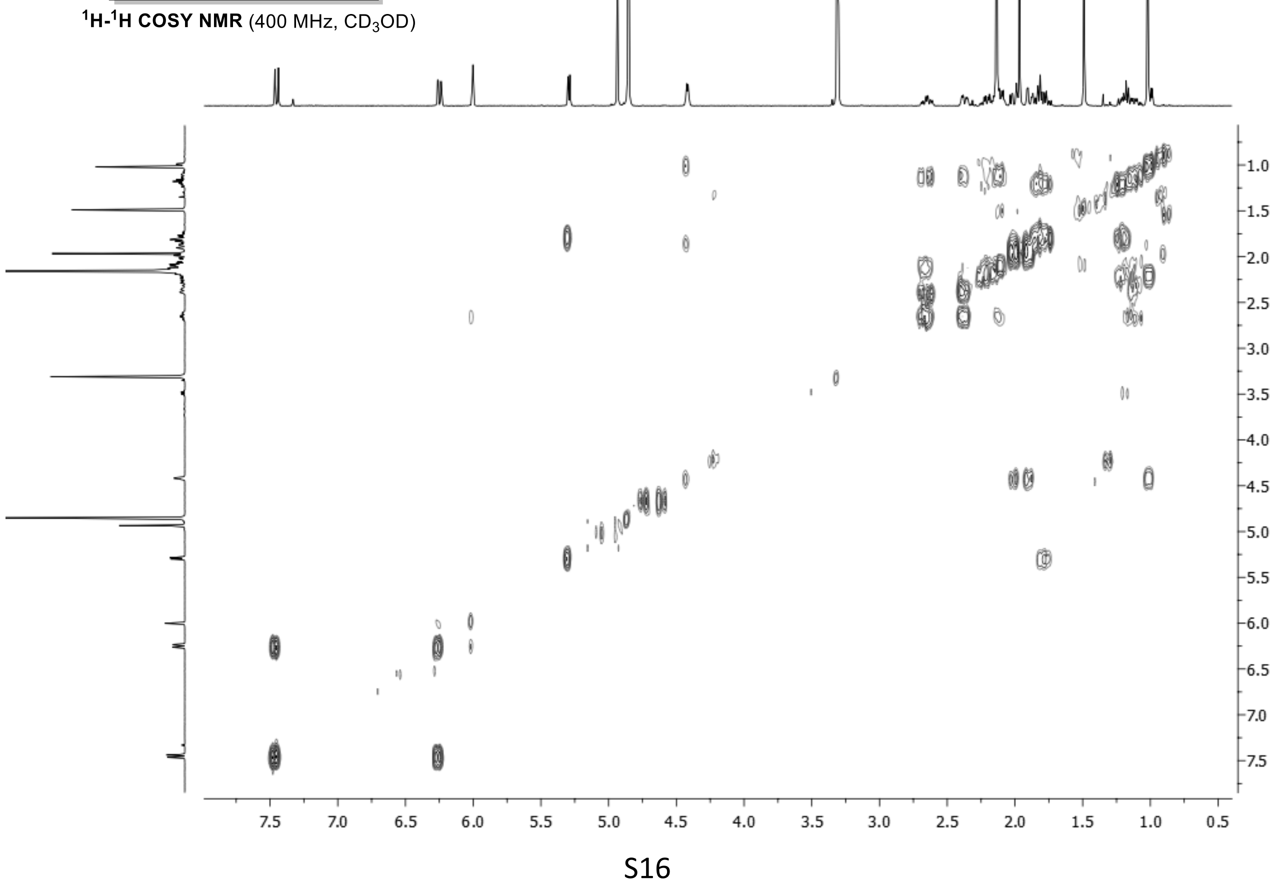

LC-MS DATA

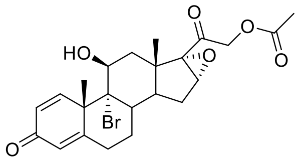

9

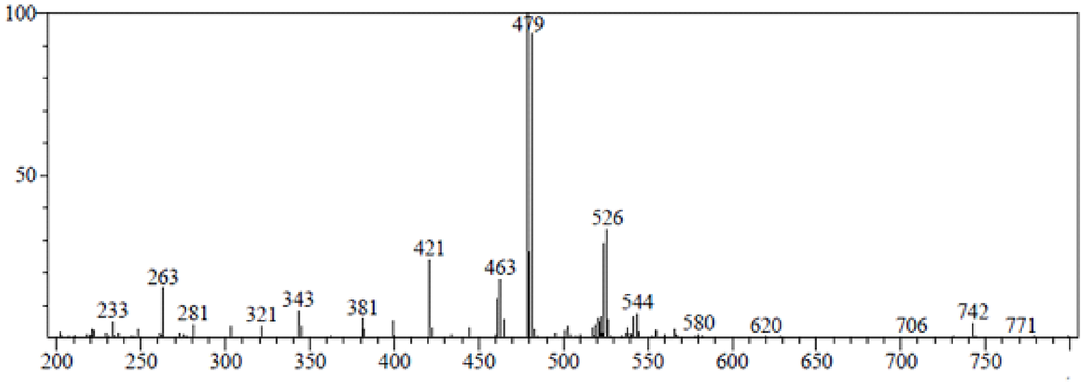

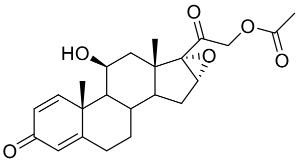

10

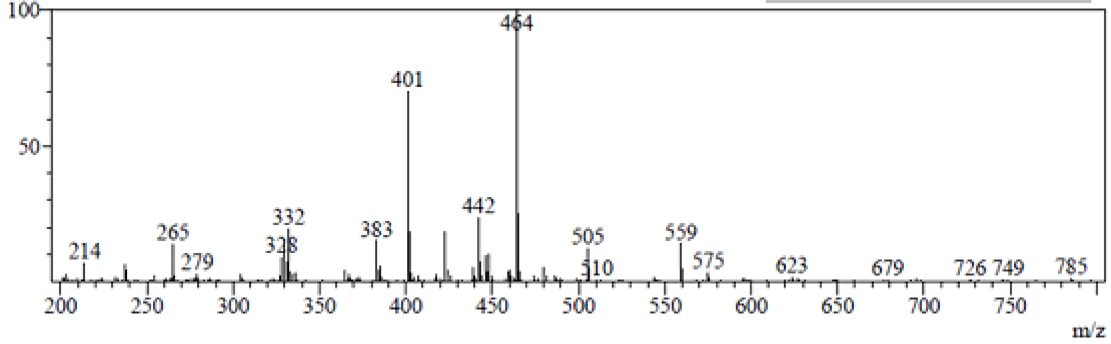

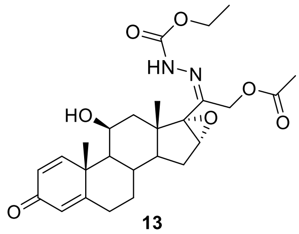

13

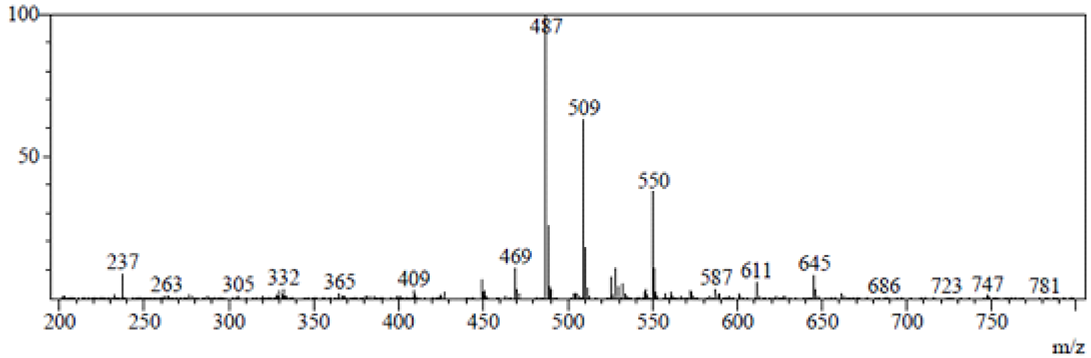

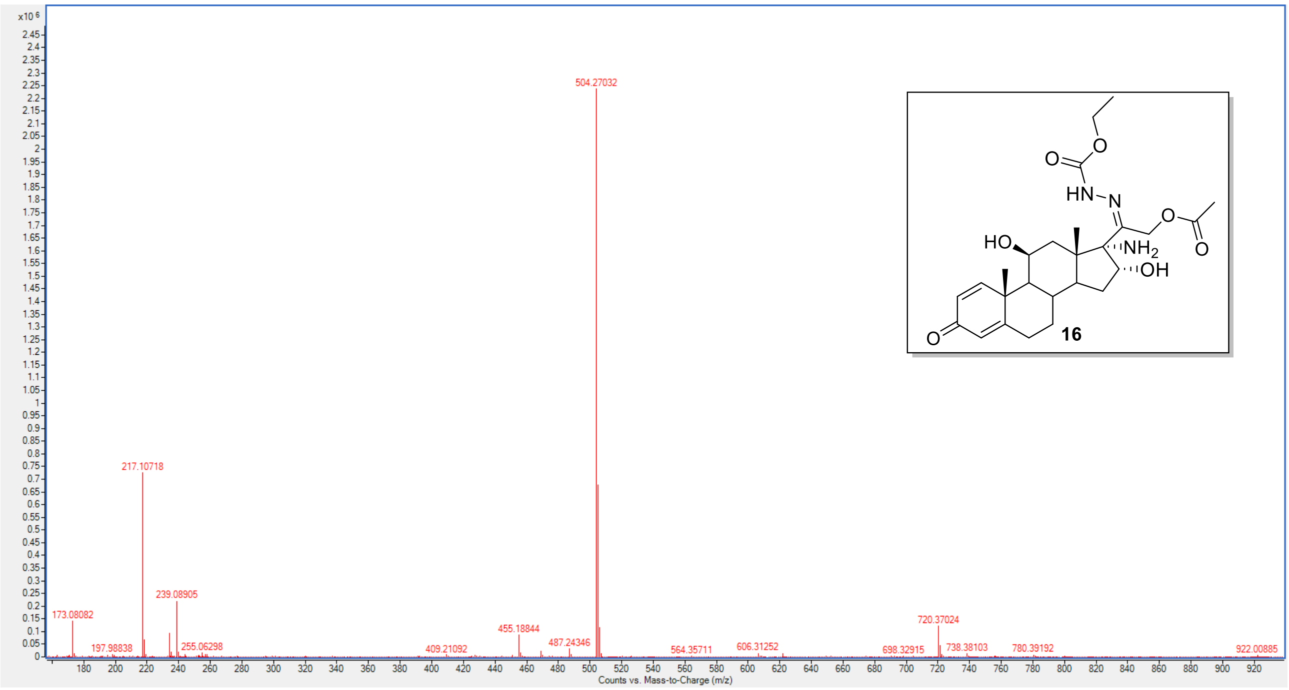
x10 6
504.27032
217.10718
239.08905
173.08082
197.98838
255.06298
409.21092
455.18844
487.24346
564.35711
606.31252
698.32915
720.37024
738.38103
780.39192
922.00885
Counts vs. Mass-to-Charge (m/z)
16

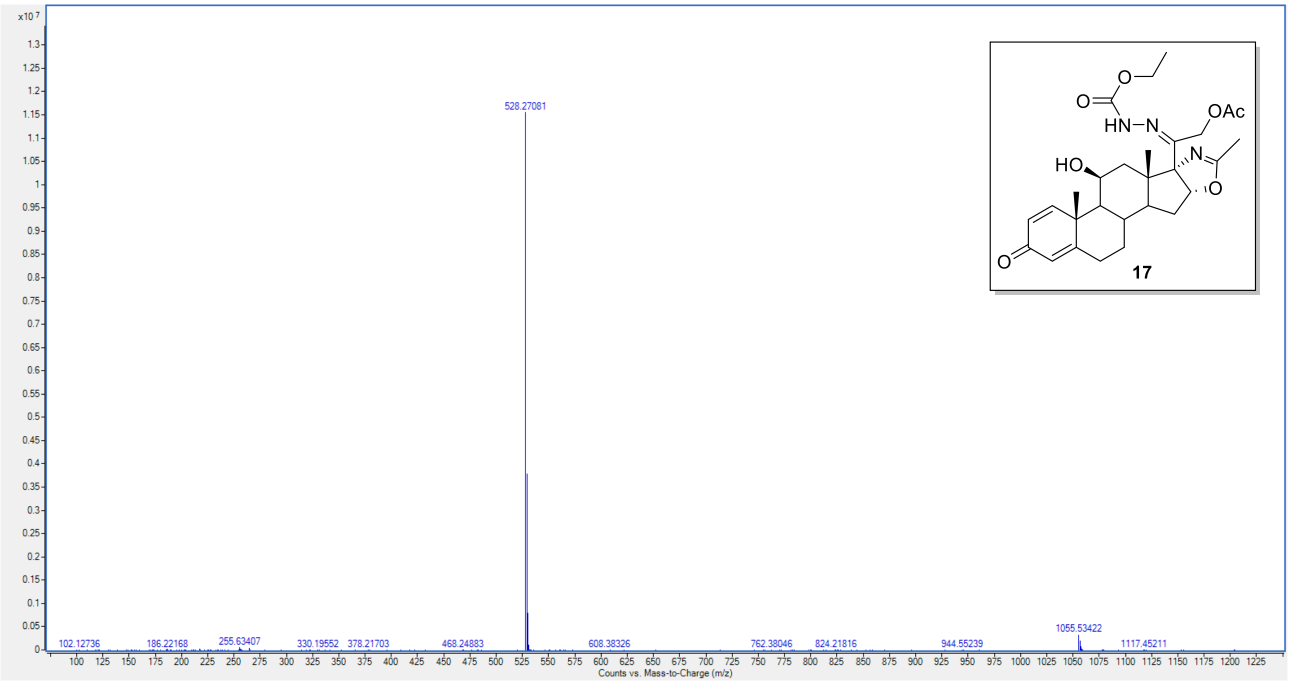
x10 7
528.27081
102.12736
186.22168
255.63407
330.19552
378.21703
468.24883
608.38326
762.38046
824.21816
944.55239
1055.53422
1117.45211
Counts vs. Mass-to-Charge (m/z)
17

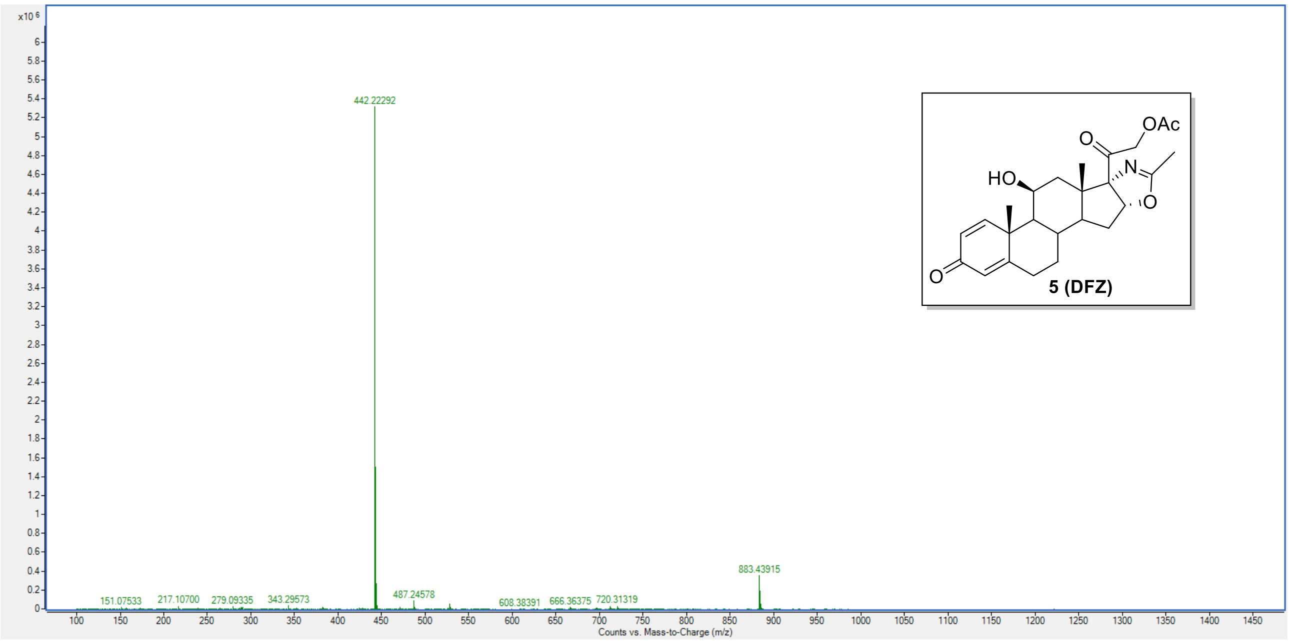
x10 6
442.22292
883.43915
151.07533
217.10700
279.09335
343.29573
487.24578
608.38391
666.36375
720.31319
Counts vs. Mass-to-Charge (m/z)
OAc
HO
N
O
5 (DFZ)

BIOLOGICAL ASSAYS

Acinetobacter baumannii ATCC 17978 and *Staphylococcus aureus* ATCC 29213, well-known as not resistant strains, were evaluated as a Gram-negative and Gram-positive model and were obtained from the American Type Culture Collection. Both strains were grown on blood agar plates (TSA), as described previously.[1] Minimum inhibitory concentration (MIC) values of steroidal compounds against planktonic bacteria were examined by a broth microdilution method previously described.[2] Briefly, compounds were dissolved in dimethyl sulfoxide (DMSO) to obtain a concentration of 50 mg/ml. Two-fold serial dilutions ranging from 1 mg/mL to 2 µg/mL of the compounds were prepared in triplicate and placed into a polystyrene 96-well plate. Bacterial cell suspensions were prepared at an equivalent to a 0.5 McFarland standard and were subsequently diluted in cation-adjusted Mueller-Hinton broth so that the final culture density was equal to 5×10^6 colony forming unit (cfu)/mL. 100 µL of bacteria (5×10^5 cfu) were then added to the microtiter plates containing steroidal compounds. One well with no antibiotic was used as a positive growth control on each plate. The plates were incubated at 37 °C for 18–24 h under shaking (300 rpm) and the MIC was calculated on the basis of concentration of compound in the well having no visible growth. To evaluate the effect of DMSO on bacteria growth kinetics, separate DMSO controls were used. To calculate the minimum bactericidal concentration (MBC), bacterial suspensions from MIC assay microtiter wells were diluted in PBS and spot-plated on TSA plates to count colonies after incubation at 37 °C for 18 h. The MBC was determined as the lowest concentration of substance, which produced ≥99.9% killing (≥3 $\log_{10}$) after 24 h of incubation as compared to the colony count of the starting inoculum. All tests were performed in triplicate and repeated three times.

REFERENCES

[1] E. De Gregorio, E. Roscetto, V.D. Iula, M. Martinucci, R. Zarrilli, P.P. Di Nocera, M.R. Catania, *New Microbiol.,* 2015; **38**, 251.

[2] K. Pane, V. Cafaro, A. Avitabile, M.T. Torres, A. Vollaro, E. De Gregorio, M.R. Catania, A. Di Maro, A. Bosso, G. Gallo, A. Zanfardino, M. Varcamonti, E. Pizzo, A. Di Donato, T.K. Lu, C. de la Fuente-Nunez, E. Notomista. *ACS Synth Biol.* 2018; **7**, 2105.
